# Degradation routes of trafficking-defective VLDLR mutants associated with Dysequilibrium syndrome

**DOI:** 10.1038/s41598-017-19053-8

**Published:** 2018-01-25

**Authors:** Praseetha Kizhakkedath, Anne John, Lihadh Al-Gazali, Bassam R. Ali

**Affiliations:** 1 Department of Pathology, College of Medicine and Health Sciences, United Arab Emirates University, Al-Ain, Abu Dhabi United Arab Emirates; 2 Department of Paediatrics, College of Medicine and Health Sciences, United Arab Emirates University, Al-Ain, Abu Dhabi United Arab Emirates; 3 Zayed Center for Health Sciences, United Arab Emirates University, Al-Ain, Abu Dhabi United Arab Emirates

## Abstract

Low density lipoprotein receptor (LDLR) family members are involved in signaling in the developing brain. Previously we have reported that missense mutations in the Very Low Density Lipoprotein Receptor gene (*VLDLR*), causing Dysequilibrium syndrome (DES), disrupt ligand-binding, due to endoplasmic reticulum (ER) retention of the mutants. We explored the degradation routes of these VLDLR mutants in cultured cells. Our results indicate that VLDLR mutants are retained in the ER for prolonged periods which could be facilitated by association with the ER-resident chaperone calnexin. The mutants were prone to aggregation and capable of eliciting ER stress. The VLDLR mutants were found to be degraded predominantly by the proteasomal pathway, since ubiquitinated VLDLR was found to accumulate in response to proteasomal inhibition. Further, the mutants were found to interact with the ER degradation adaptor protein SEL1L. The degradation of VLDLR wild type and mutant were delayed in CRISPR/Cas9 edited SEL1L knock-out cells which was reversed by exogenous expression of SEL1L. In summary, ER retention of pathogenic VLDLR mutants involves binding to calnexin, elevated ER stress, and delayed degradation which is dependent on SEL1L. Since core LDLR family members share common structural domains, common mechanisms may be involved in their ER processing.

## Introduction

Low density lipoprotein receptor (LDLR) family members are important mediators of signaling events during brain development and defects in many of these receptors relate to distinct neurobehavioural phenotypes^1^. All are expressed in the developing and/or the adult brain and common structural domains are the hallmark of all core members^2^. Dysequilibrium syndrome (DES, OMIM 224050) is an autosomal recessive disorder and a subgroup of this disorder has been associated with mutations in the gene encoding the very low density lipoprotein receptor (VLDLR)^3–5^. VLDLR is a multi-ligand receptor belonging to the low density lipoprotein receptor (LDLR) family and is highly expressed in brain, heart, and skeletal muscle tissues^6^. In neurons, VLDLR is a receptor for Reelin and an integral part of the Reelin signaling pathway that directs the migration of neuroblasts during embryonic brain development^7^. Previously we have reported that three missense mutations in VLDLR (c.1459 G > T; p.D487Y, c.1561 G > C; p.D521H and c.2117 G > T; p.C706F), associated with DES, inhibit the cell surface trafficking of the receptor and thus interrupt binding to its ligand Reelin^8^.

Transport of newly synthesized proteins in ER through the secretory pathway is strictly regulated by a quality control system (ERQC) which ensures that only properly folded and assembled proteins exit ER while misfolded and misassembled proteins are retained in the ER^9^. Misfolded proteins that fail to conform to the ERQC are eventually dislocated into the cytosol and degraded by the proteasome by a process termed as ER-associated protein degradation (ERAD)^10^. Membrane-embedded ubiquitin-ligase containing complexes orchestrate the extraction and ubiquitination of misfolded proteins in ER which are subsequently delivered to the proteasome. HRD1/SEL1L complex is one such complex central to mammalian ERAD^10^. ERAD dysfunction results in chronic ER stress which is a pathological hallmark of several diseases^11^. Prolonged ER stress induced by misfolded proteins cause initiation of macroautophagy programs termed ER-activated autophagy (ERAA) pathway, which serves to suppress ER stress and prevent cell death^12^. In certain instances, misfolded membrane and ER lumenal proteins that form aggregates and place constraints on ER translocation machinery are diverted to the lysosome for degradation via autophagy^13^

The accumulation of unfolded proteins in the ER lumen also activates an adaptive response program in the cell, termed as the unfolded protein response (UPR), which acts in concert with ERAD to eliminate ER stress and achieve ER homeostasis^14^. In mammals, inositol-requiring enzyme 1α (IRE1α) initiates the most conserved UPR signaling pathway. Activated IRE1α catalyzes the unconventional splicing of the mRNA of the transcription factor X-box binding protein-1, which in turn targets the regulation of a class of UPR-related genes that are involved in protein folding, protein entry to the ER, and ER-associated degradation (ERAD)^14^.

Mutations in the closely related receptor LDLR, including those corresponding to the pathogenic mutations reported in VLDLR, primarily account for the hereditary disorder familial hypercholesterolemia (FH)^15^. Approximately 50% of the LDLR mutations are class II mutations that cause the mutant protein to be retained in the endoplasmic reticulum (ER) and ER-associated proteasomal degradation (ERAD) has been reported to be the principal pathway of degradation of these mutants^16^. It has also been reported that LDLR mutants implicated in FH, cause ER stress and activation of UPR pathways^17^. Chemical chaperones like glycerol and 4-PBA have been able to restore functionality of some of the LDLR class 2 receptors in a mutation-specific manner^18^, which can be explored for manipulating the disease outcomes caused by similar mutations in other lipoprotein receptors, given their structural similarity.

We hypothesized that mutations affecting identical conserved residues of the two low density lipoprotein receptors (VLDLR and LDLR) will predispose the mutant proteins for similar cellular fates. Hence the ER-retained VLDLR mutants could be potential substrates of the ERAD pathway. Our results indicated that VLDLR mutants are not subjected to premature degradation in the ER, but retained in the ER for long periods of time, which could be facilitated by interactions with the ER- resident chaperone calnexin. The eventual disposal of the mutants appear to depend primarily on the ubiquitin-proteasome pathway, since ubiquitinated aggregates were found to accumulate more under conditions of proteasomal inhibition rather than lysosomal inhibition. We also report that the ERAD adaptor protein SEL1L interact with VLDLR and by CRISPR/Cas9 functional knock-out, provide evidence for a role for SEL1L in the intracellular degradation of VLDLR.

## Results

### VLDLR mutants have slower turn-over rates than the wild type receptor

**Figure 1:**
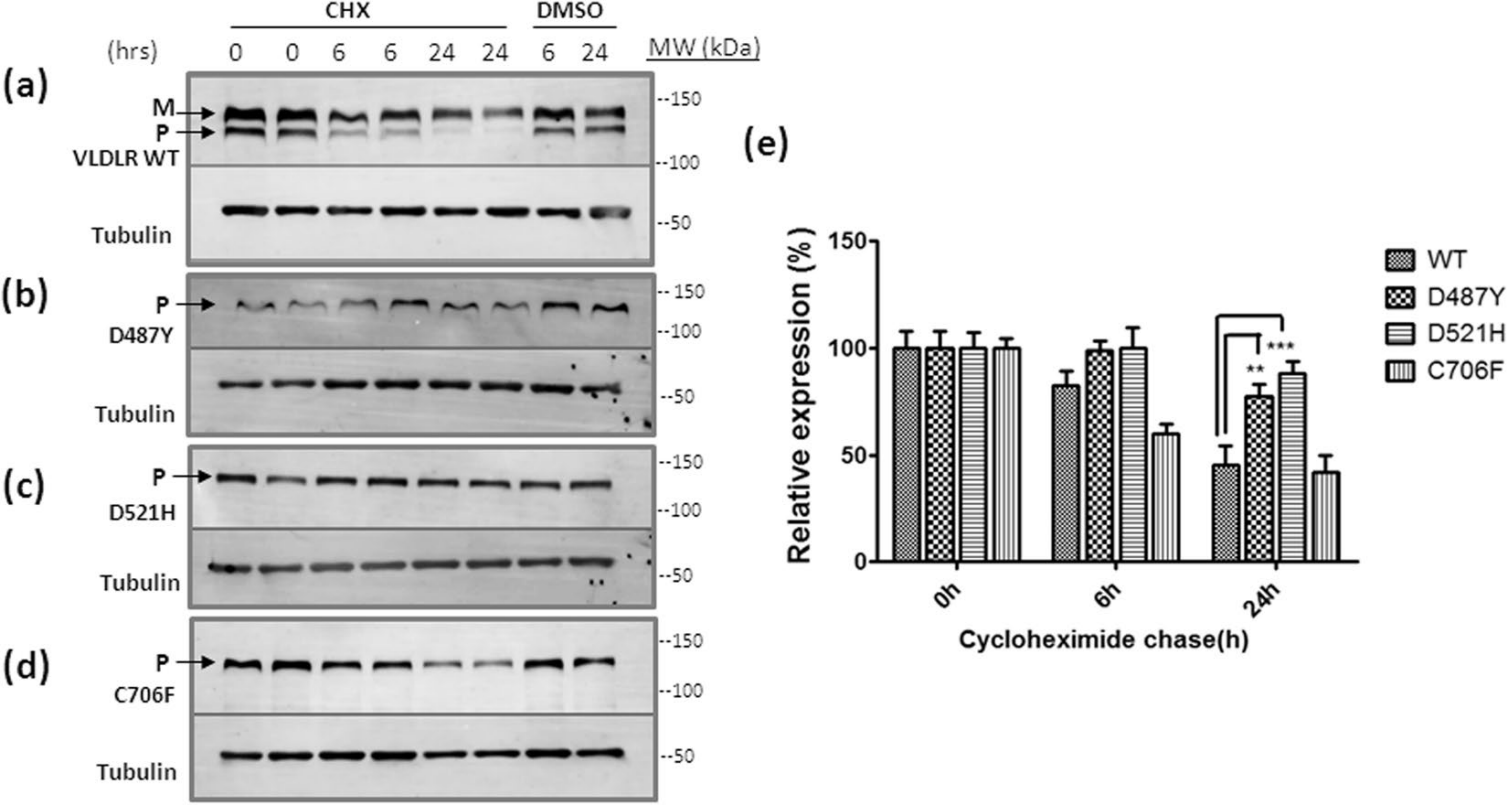
Cycloheximide (CHX) chase assays showing slower turn-over rates of VLDLR mutants than the wild type. HEK-293 cells were transfected with the indicated VLDLR-HA plasmids. At 24 h after transfection, the cells were incubated with 100 µg/ml CHX or DMSO for 24 h and collected at different times (6 h & 24 h) for western blot. Independent replicates for each time point are shown in the blots. Immunoblots (IB) were probed with anti-HA antibody. Alpha-tubulin was used as a loading control. The mature (M) and precursor (P) forms of VLDLR are indicated by arrows. Tubulin normalized VLDLR protein levels at 0 h were defined as 1.0 for each panel. **(a)** VLDLR-WT**, (b)** VLDLR-D487Y, **(c)** VLDLR-D521H, **(d)** VLDLR-C706F, **(e)** Densitometric analysis of time-dependent degradation of VLDLR WT versus mutants: Error bars represent ± S.E.M. of three experiments, (**); *p* ≤ 0.01; (***) *p* ≤ 0.001; Two-way ANOVA; n = 5. Regions cropped from separate images are demarcated with borders. Unprocessed original scans of western blots are shown in Supplementary Figure S8.

Previously we have reported that missense mutants of the VLDLR (D487Y, D521H and C706F) receptor were transport deficient and dysfunctional^8^. To analyze the possibility of increased intracellular degradation of VLDLR mutants, turn-over rates of the three missense mutants, D487Y, D521H, C706Y and wild type VLDLR (WT) receptor were determined by cycloheximide translation shut-off assay in HEK-293 cells overexpressing the wild type or mutants. In immunoblots VLDLR run as two distinct bands, the fully glycosylated mature form (150 kDa) and the precursor form in transit in ER (130 kDa). The precursor form matures into the fully glycosylated form within 6 h and the mature wild type VLDLR receptor has been reported to have a half-life of ~24 h^19^. We have reported previously that none of the three missense mutants analyzed in this study, attain Endoglycosidase H resistant advanced glycosylation specific to Golgi^8^. After 24 h of cycloheximide chase, only the mature glycosylated form was visible in the immunoblots of wild type (Fig. 1a) and the total VLDLR levels were reduced to ~50%. On the other hand, the precursor forms of the mutants D487Y and D521H were found to have slower degradation rates than the wild type (Fig. 1b,c & d). After 24 h of cycloheximide chase the relative expression levels of the mutants D487Y and D521H were found to vary significantly from that of wild type, while the mutant C706F protein levels were similar to that of the wild type (Fig. 1e). Thus the ER retention of the VLDLR mutants does not lead to their premature intracellular degradation. This could be due to prolonged interaction with ER chaperones or formation of degradation resistant aggregates.

### VLDLR mutants are prone to aggregation and cause ER stress

**Figure 2:**
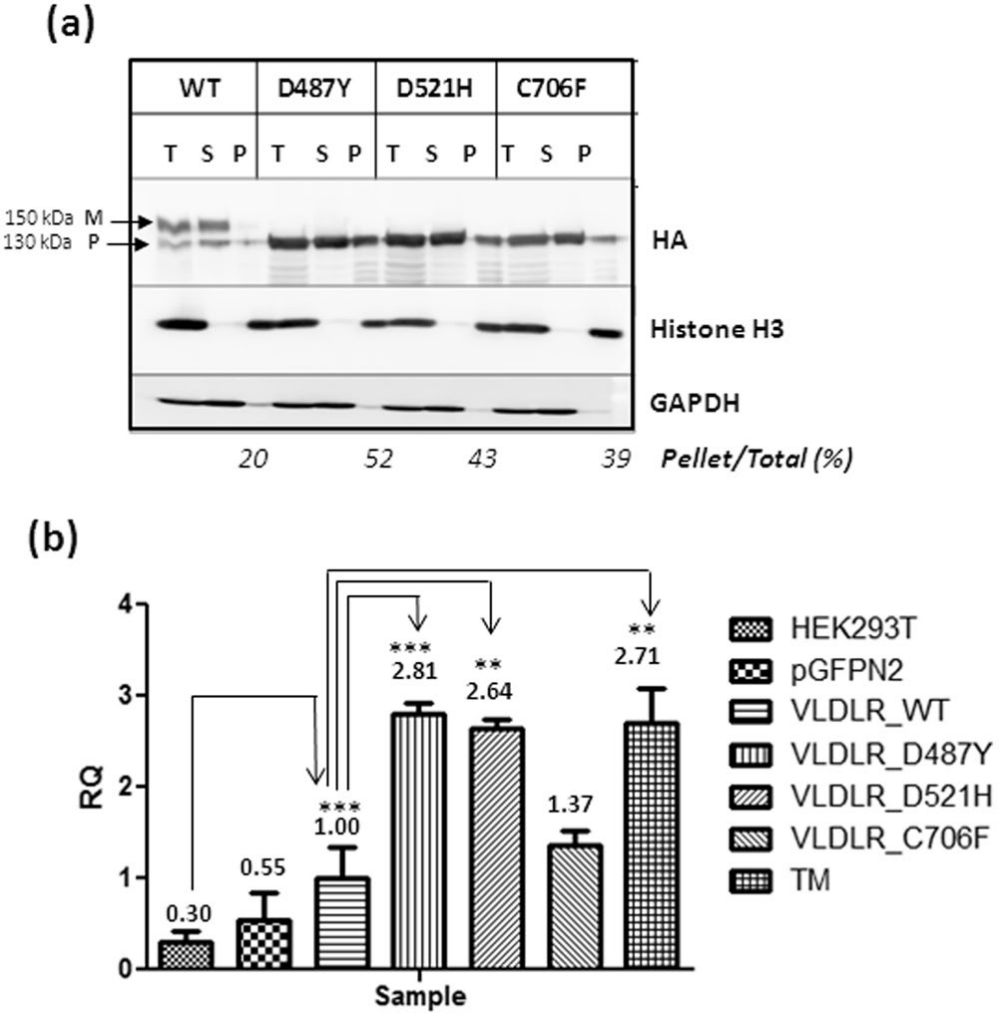
Analysis of aggregation states of VLDLR mutants and measurement. of ER stress **(a)** Analysis of VLDLR solubility in the nonionic detergent Triton X-100. HEK-293T cells were transiently transfected with the indicated plasmids. Cell extracts were prepared in lysis buffer supplemented with 1% Triton X-100 and centrifuged at 4 °C at 20,000 × g for 15 min. The total cell lysate (T), pellet (P), and supernatant (S) fractions were analyzed for presence of respective VLDLR proteins by western blot against HA. The mature (M) and precursor (P) forms of VLDLR are indicated by arrows Histone H3 and GAPDH were used as controls for pellet and soluble fractions respectively. The experiment was performed twice with similar results. Regions cropped from separate images are demarcated with borders. Unprocessed original scans of blots are shown in Supplementary Figure S9. **(b)** Induction of ER stress in HEK-293T cells 48 h post transfection with VLDLR WT or mutants. Aggregation-prone VLDLR mutants induce ER stress, represented through elevated alternatively spliced XBP-1 transcript levels, measured through quantitative PCR (qPCR). The XBP-1s mRNA levels of the VLDLR WT at 48 h post-transfection was set as 1.00. Fold-changes mRNA expression of the mutants were expressed in relation to WT. Error bars represent ± S.E.M. of three experiments; (*) *p* ≤ 0.05; (**) *p* ≤ 0.01; (***) *p* ≤ 0.001; One-way ANOVA, Bonferroni post-test.

To analyze whether the ER- retained VLDLR mutants form insoluble aggregates, the aggregation status of VLDLR wild type and mutants were analyzed by Triton X-100 solubility assay in HEK-293T and HeLa cells transiently over-expressing the same. The VLDLR wild type and a significant proportion of mutants were predominantly detected in the soluble fraction (Fig. 2a). However, a fraction of the mutants were found in the insoluble pellet fraction. The mutants D487Y and D521H were found to be more prone to aggregation than the C706F mutant. We next examined if the mutants exert ER stress and unfolded protein response by assessing the expression levels of spliced XBP-1 mRNA. Tunicamycin-treated cells were used as a positive control for ER-stress induction. In the cells expressing the wild type receptor, XBP-1s transcript levels were comparable to that of cells expressing GFP indicating that exogenous over expression of the wild type receptor did not affect ER function. The fold-changes in XBP-1s mRNA levels in cells expressing aggregation-prone mutants were greater than 2-fold than the wild-type expressing cells and were found to be statistically significant (Fig. 2b). The XBP-1s expression was more pronounced in the cells expressing the relatively aggregation-prone mutants D487Y and D521 and was found to be induced as early as 24 h (Supplementary Figure S1).

### Degradation of VLDLR mutants is dependent predominantly on the proteasomal pathway

**Figure 3:**
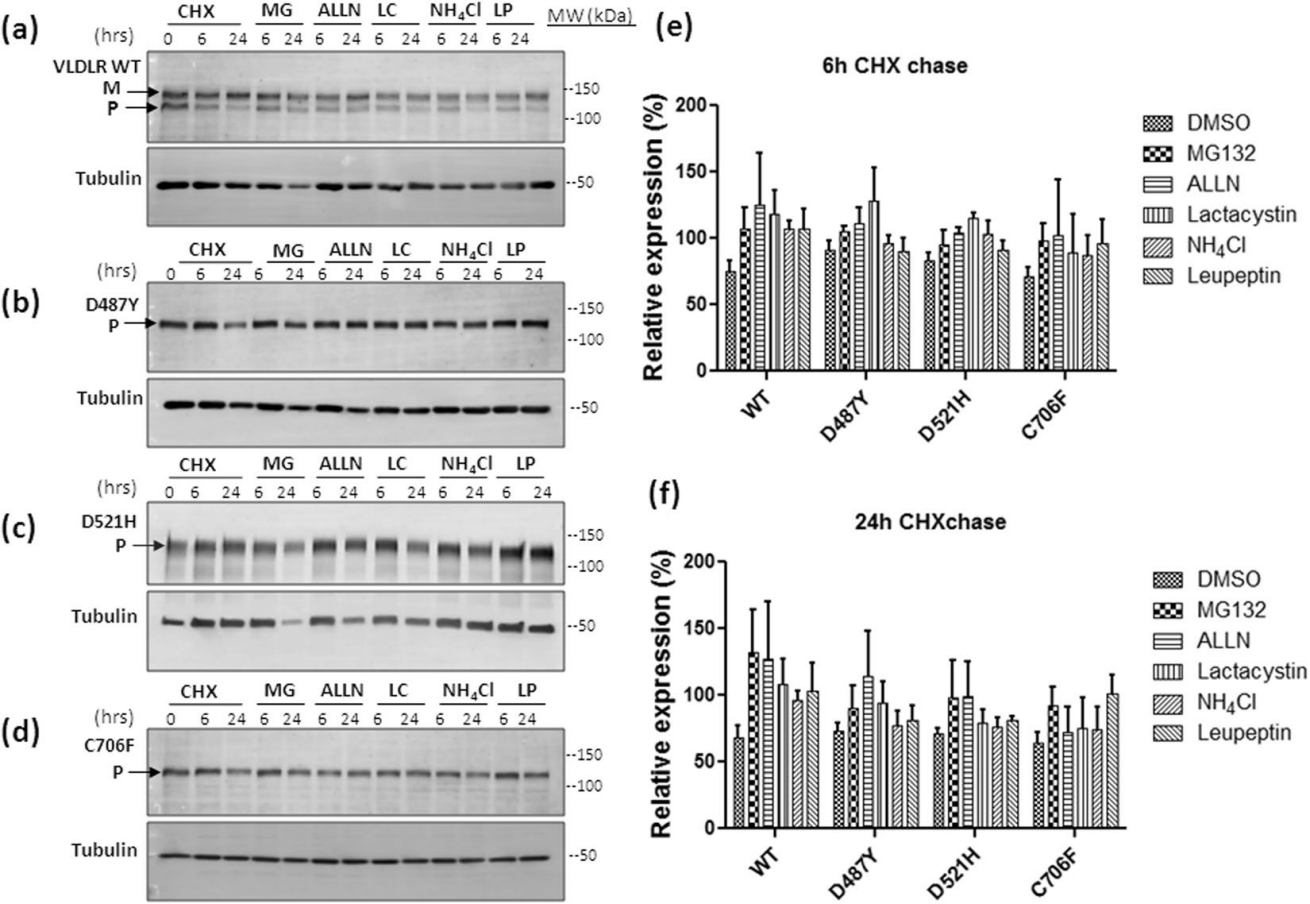
Effect of proteasomal or lysosomal inhibitors on the degradation rates of VLDLR WT and mutants. HEK-293 cells transiently expressing the VLDLR WT (**a**) or mutants (**b**–**d**) mutants were treated with cycloheximide (CHX) in the presence of either inhibitors of proteasome (10 µM MG132, 10 µM ALLN or 10 µM lactacystin) or lysosome (0.1 mM leupeptin or 20 mM NH_4_Cl) as indicated. Total cell lysates were analyzed by immunoblotting against HA. Relative amounts of respective proteins remaining at the indicated time points were quantified, and normalized to tubulin levels. Tubulin normalized VLDLR protein levels at 0 h were defined as 1.0 for each panel. The experiments were performed thrice with identical results. (**e**) Graph representing the relative mean densities of vehicle (DMSO) or inhibitor treated wild type VLDLR and all the mutants at 6 h time-point (**f**) Graph representing the relative mean densities of DMSO or inhibitor treated VLDLR wild type and all the mutants at 24 h time-point. Error bars represent SEM from n = 3 independent experiments. Regions cropped from separate images are demarcated with borders. Unprocessed original scans of western blots are shown in Supplementary Figure S10.

To investigate the degradation pathways of these mutants, cells overexpressing the wild type and mutant receptors were treated with various proteasomal and lysosomal inhibitors prior to cycloheximide chase. Increase in total ubiquitinated proteins in lysates from inhibitor-treated cells was taken as an evidence for inhibition of proteasome function (Supplementary Figure S2a). Accumulation of the autophagy reporter LC3B was taken as evidence for lysosomal inhibition (Supplementary Figure S2b). The expression level of the wild type VLDLR was found to be sensitive to inhibition with all the proteasomal (MG132, ALLN and lactacystin) and lysosomal inhibitors (NH_4_Cl, leupeptin). At 6 h the VLDLR WT expression was found to be sensitive to inhibition with both proteasomal and lysosomal inhibitors (Fig. 3a). The expression levels of the mutant D487Y was found to be sensitive to all the proteasomal inhibitors (Fig. 3b) at 6 h and 24 h of treatment. Half-life of the mutant D521H was sensitive to inhibition with all the proteasomal and lysosomal inhibitors for short-term treatment period (6 h) (Fig. 3c). Upon treatment for a longer period (24 h) the expression level of this mutant was found to be stabilized mainly by proteasomal inhibition. Only moderate differences were observed in the expression levels of the mutants D487Y and D521H in response to various inhibitors and this could be due to the slower degradation rates of these mutants.

Expression levels of the mutant C706F was stabilized by the proteasomal inhibitor MG132 and lysosomal inhibition with leupeptin for short-term and prolonged treatment (Fig. 3d). The differences in protein expression levels before and after treatment with inhibitors were more evident for this mutant, probably due to its faster degradation rate. To confirm the results, the effect of more than one proteasomal (MG132 + Lactacystin) or lysosomal inhibitors NH_4_Cl + Leupetin) were examined on the half-lives of either wild type or the mutant C706F with an increased concentration of cycloheximide (150 µg/µl). Under these conditions, differences in the expression levels of the WT and mutant were clearly evident at 6 h (Supplementary Figure S3).

**Figure 4:**
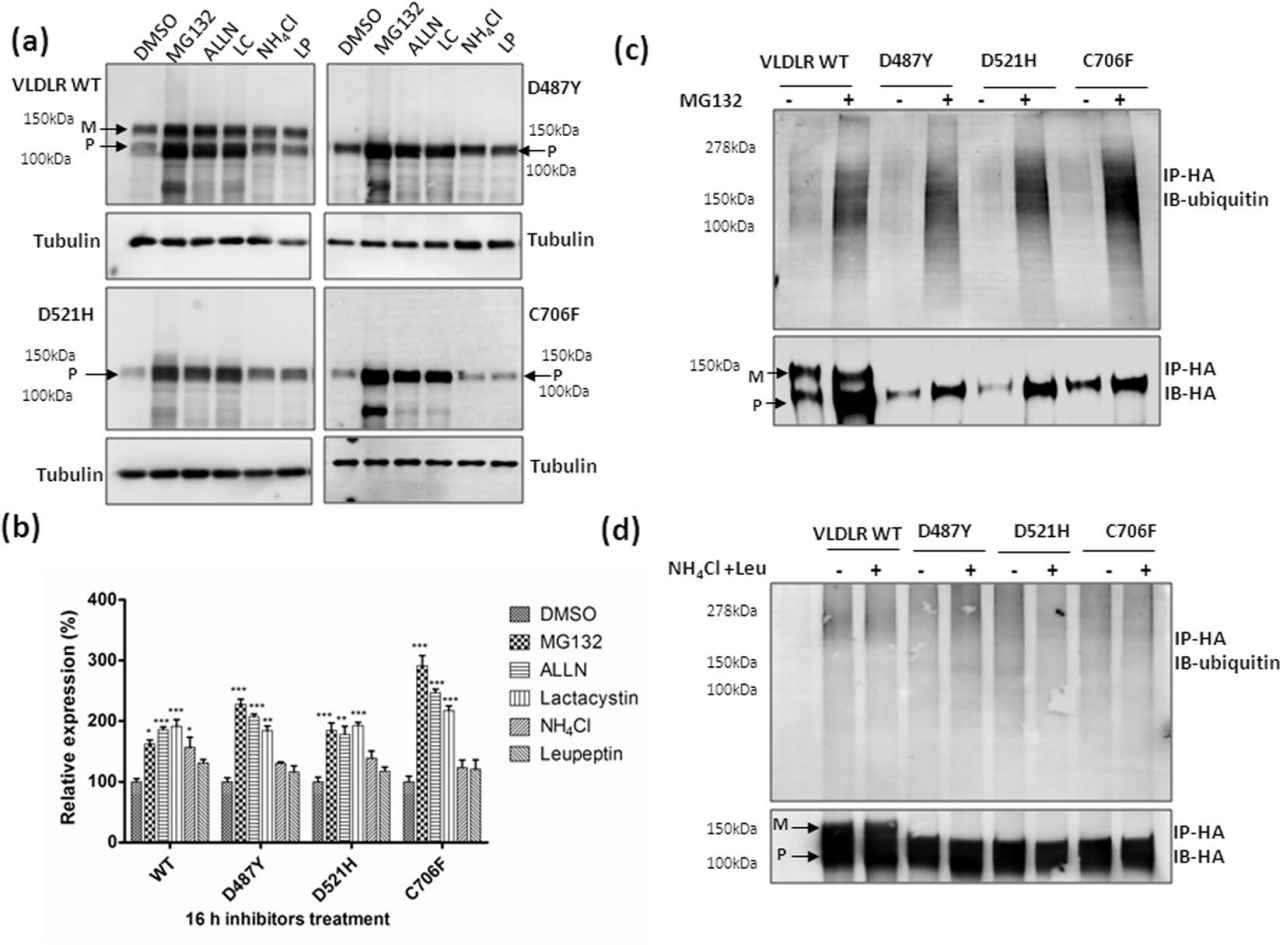
Accumulation of ubiquitinated species of VLDLR WT or mutants in response to proteasomal and lysosomal inhibition: **(a)** HEK-293T cells transiently expressing VLDLR WT or mutants (D487&, D521H& C706F) were treated with DMSO(−) or either inhibitors of proteasome (10 µM MG132, 10 µM ALLN or 10 µM lactacystin (LC)) or lysosome (0.1 mM leupeptin (LP) or 20 mM NH_4_Cl) for 16 h and total cell lysates were analyzed by western blotting against HA. Tubulin was used as loading control. **(b)** Graph showing relative mean densities of WT and mutants in response to different inhibitors. Tubulin normalized VLDLR protein levels of DMSO treated samples were defined as 1.0. (**); *p* ≤ 0.01; (***) *p* ≤ 0.001; Two-way ANOVA, Bonferroni post-test; n = 3. **(c)** Total VLDLR was immunoprecipitated (IP) using HA-agarose from HEK-293T cells transfected with VLDLR WT or mutants and treated with 10 µM MG132 (+) or DMSO (−) for 16 hrs. The immunoprecipitates (IP) were immunoblotted with antibodies against Ubiquitin or HA. This experiment was performed thrice with identical results. (**d**) Total VLDLR was immunoprecipitated from cells expressing the indicated constructs and treated with lysosomal inhibitors (0.1 mM leupeptin (LP)+20 mM NH_4_Cl) for 16 h. The IP fractions were probed against Ubiquitin or HA. The experiments were performed twice with identical results. Regions cropped from separate images are demarcated with borders. Unprocessed original scans of western blots are shown in Supplementary Figure S11.

When cells overexpressing the wild type and mutant receptors were treated with proteasome inhibitors MG132, ALLN or lactacystin alone for longer periods (16 h), there was several-fold accumulation in the wild type as well as mutant protein levels compared to untreated (Fig. 4a & b). In addition, accumulation of higher molecular weight ubiquitinated forms was observed in both VLDLR WT and mutants (Fig. 4c) in response to MG132. Maximum protein accumulation of the mutants was observed in response to MG132 treatment than with more specific inhibitors ALLN or lactacystin. Treatment (16 h) with the lysosomal inhibitors alone did not appear to enhance the protein levels of the mutants significantly. This was intriguing especially since the mutant protein levels (D521H and C706F mutants) were stabilized by either short-term or long-term treatment with leupeptin in the presence of cycloheximide. It had been reported that, simultaneous administration of cycloheximide with leupeptin blocks the formation of autophagic vacuoles by possibly blocking the synthesis of some protein or peptide essential for the autophagic process^20,21^. Therefore, it is possible that some nonspecific autophagic pathway is involved in the degradation of these mutants, which requires further investigation. The accumulation of ubiquitinated wild type and mutant receptors in response to long-term treatment with lysosomal inhibitors, NH_4_Cl and leupeptin for 16 h, was not as striking as that observed with MG132 (Fig. 4d). Since ubiquitinated mutant VLDLR was found to accumulate significantly when the proteasome was blocked, it could be possible that ERAD is the primary degradation pathway for the mutants and a pool of ERAD resistant mutants form aggregates and are disposed of by autophagy.

The wild type VLDLR also accumulated in MG132, ALLN or lactacystin-treated cells, but the increase was observed predominantly for the precursor form. The cell surface localized VLDLR WT is reported to be degraded by the lysosomal pathway^22^. But exogenously expressed VLDLR WT have been reported to be stabilized by MG132^23^. Our results also indicate increase in the levels of mature form of VLDLR WT in response to lysosomal inhibitors. The half-life of the VLDLR precursor is reported to be longer than that of the LDLR and in stable expressing cells, only 70% of the precursor gets converted to the mature form^24^. It is possible that a fraction of the newly synthesized wild type protein is degraded by the proteasomal pathway.

### VLDLR mutants interact with ER quality control components

**Figure 5:**
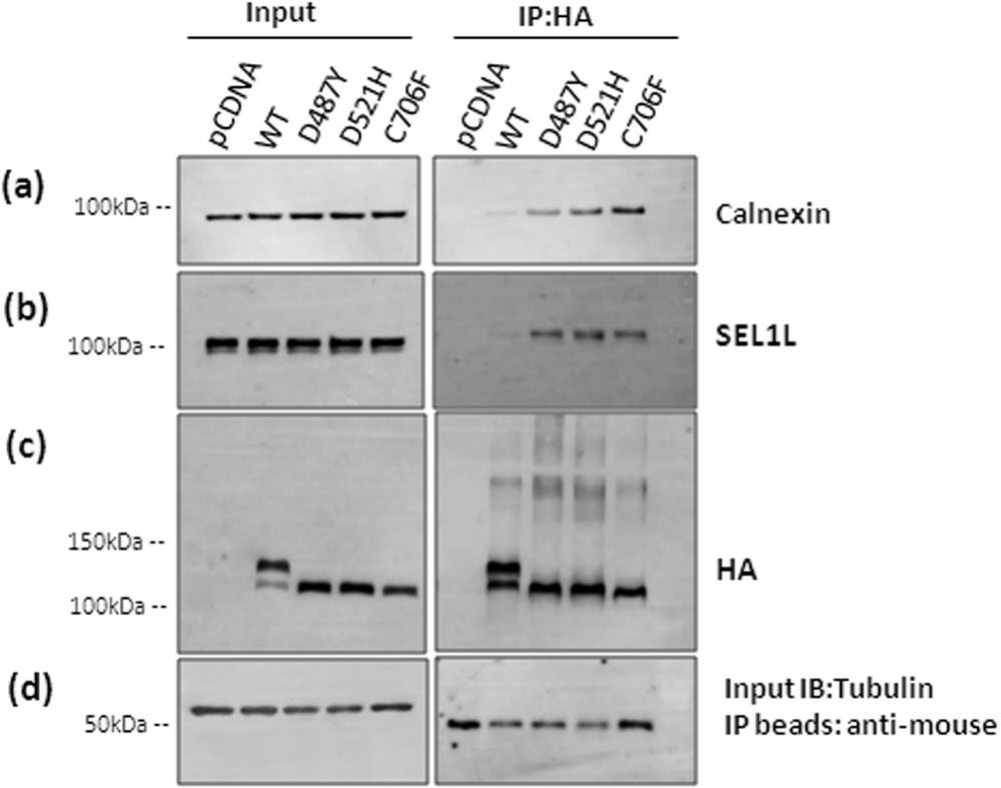
Association of Calnexin and ERAD adaptor protein SEL1L with VLDLR WT and mutants: Cell lysates were prepared from transiently transfected cells expressing either wild-type or mutant VLDLR-HA (D487Y, D521H and C706F). Cell lysates (200 µg) were subjected to co-immunoprecipitation (Co-IP) using anti-HA agarose beads. Western blot analysis of the immunoprecipitated complexes were performed using antibodies against calnexin (**a**), SEL1L (**b**) and HA (**c**). Tubulin was used as a loading control for input (**d**) and IP beads probed against anti-mouse-HRP served as loading control for IP (**d**). The experiments were performed twice for each construct with identical results. Regions cropped from separate images are demarcated with borders. Unprocessed original scans of western blots are shown in Supplementary Figure S12.

To get more clarity on whether VLDLR mutants are retained in the ER due to prolonged interaction with any of the ER quality control factors, cell lysates from HEK- 293T cells expressing the wild type or mutants were subjected to immunoprecipitation under non-denaturing conditions. Since the VLDLR mutants were not rapidly degraded in the cells, it is possible that they are trapped in unproductive calnexin/calreticulin binding cycles. The immunoprecipitates were probed against calnexin, which is reported to play a central role in the retention machinery contributing to glycoprotein quality control. Calnexin co-immunoprecipitated with wild type as well as mutant VLDLRs (Fig. 5a). In the wild type co-immunoprecipitate, the calnexin signal was very faint, probably representing a transient and productive interaction. On the other hand, all the three mutants showed a strong association with calnexin as evident from the immunoblots (Fig. 5a). No cross-linker was used during immunoprecipitation and this suggests a strong association of mutants with calnexin. Even though the soluble fractions were subjected to immunoprecipitation, higher molecular weight aggregates were visible in the VLDLR wild type as well as mutants, more so in the D487Y and D521Y mutants, in immunoblots against HA (Fig. 5c). Our results indicate that VLDLR mutants are probably retained in the ER due to prolonged association with Calnexin.

We also examined the interaction of VLDLR mutants with ERAD factors OS-9, HRD1 and SEL1L. Only SEL1L co-immunoprecipitated with VLDLR wild type and mutants, though a faint SEL1L signal was observed in VLDLR WT immunoprecipitates (Fig. 5b). All the three mutants showed strong association with SEL1L. No association with OS-9 and HRD1 was evident from immunoblots against these proteins. It is possible that interactions of these components with mutants are transient in nature and could not be captured without the use of a cross-linker. Moreover, it was reported earlier that SEl1L loses its interaction with HRD1 complex in NP-40^25^.Yet it is intriguing that the mutants form stable complexes with SEL1L. VLDLR mutants are membrane-anchored proteins with defects in the luminal domain. It was reported earlier that SEL1L has stringent requirement for engaging clients and only misfolded lumenal proteins associate with SEL1L^26^. Apart from a few endogenous transmembrane proteins that are part of the UPR, only limited literature is available on membrane-tethered proteins with misfolded lumenal domains that associate with SEL1L.

### SEL1L deficiency enhances the stability of VLDLR wild type and mutants

**Figure 6:**
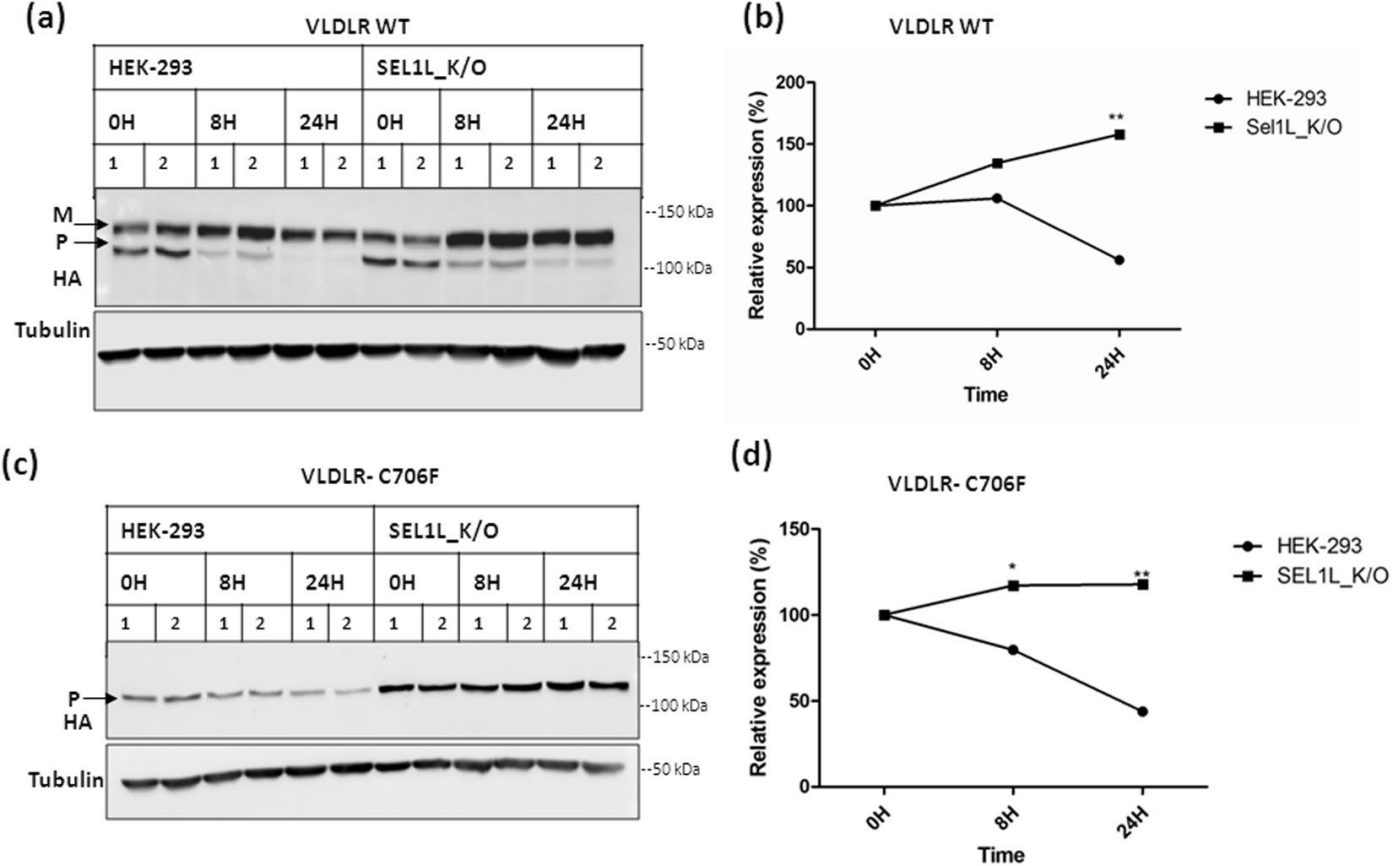
Delayed degradation of VLDLR WT and mutant in SEL1L knockout cells. VLDLR WT or the mutant C706F was transfected in HEK-293 cell lines and SEL1L Knockout (K/O) cell lines (generated by gRNA1) in parallel. At 24 h after transfection, the cells were incubated with 100 µg/ml CHX for 24 h and collected at different times (8 h & 24 h) for western blot. Replicates for each time point were analyzed in the same blot. The blots were probed against HA and tubulin. Tubulin normalized VLDLR protein levels at 0 h were defined as 100%. **(a)** Immunoblots showing the turn-over of VLDLR-WT protein. **(b)** Densitometric analysis of three independent experiments are reported in the graph. (*) *p* ≤ 0.05; (**); *p* ≤ 0.01; (***) *p* ≤ 0.001; Student’s *t*-test. **(c)** Turn-over rates of VLDLR C706F in HEK-293 cells and SEL1L knock-out cell lines. **(d)** Graph representing densitometric analysis of three independent experiments. *p* values as indicated in (**b**). Regions cropped from separate images are demarcated with borders. Unprocessed original scans of western blots are shown in Supplementary Figure S13.

The HRD1-SEL1L complex is an evolutionarily conserved membrane complex central to mammalian ERAD and has been reported to participate in the degradation of secreted proteins with misfolded lumenal domains^26^. Depletion of SEL1L has been reported to destabilize HRD1 and prevent the degradation of misfolded lumenal/transmembrane proteins^27–29^. Accumulation of ubiquitinated mutant protein in response to proteasome inhibition and interaction with the ERAD adaptor protein SEL1L suggest that the VLDLR mutants are degraded by the ubiquitin- proteasome system through the HRD1/SEL1L complex. To gain more clarity on the role of SEL1L in the quality control of VLDLR mutants, we studied in detail the effect of SEL1L on VLDLR WT and one of the mutants C706F. The mutant C706F was selected for further studies because the degradation rate of this mutant was comparable to that of the wild type. The turn-over rates of VLDLR WT and the mutant were analyzed in SEL1L knock-out cell lines generated by CRISPR/Cas9 gene editing. Disruption of SEL1L in knock-out cells was confirmed by immunoblotting against SEL1L, HRD1 and OS-9. As expected SEL1L deficiency destabilized ERAD complex as evidenced by reduced expression of HRD1 and accumulation of OS-9 (Supplementary Figure S4). The half-lives of VLDLR wild type and C706F mutant were examined by transient transfection and translational shut-off by cycloheximide. The wild type and mutant showed steady degradation after 24 h of cycloheximide treatment in HEK-293 cells (Fig. 6a & b). In SEL1L knockout cell lines, the steady-state levels of the wild type as well as the C706F mutant remained the same even after 24 h of cycloheximide chase. To confirm that the observed results were not due to off-target effects of the gRNA used for gene editing, the experiments were repeated in another knock-out cell line generated by a different gRNA. Similar results were obtained in the second SEL1L knock-out cell line also, though the stabilization of wild type was not as pronounced as in the first cell line (Supplementary Figures S4 and S5).

### Exogenous expression of SEL1L in knock-out cell lines restores the degradation of wild type and mutant VLDLR

**Figure 7:**
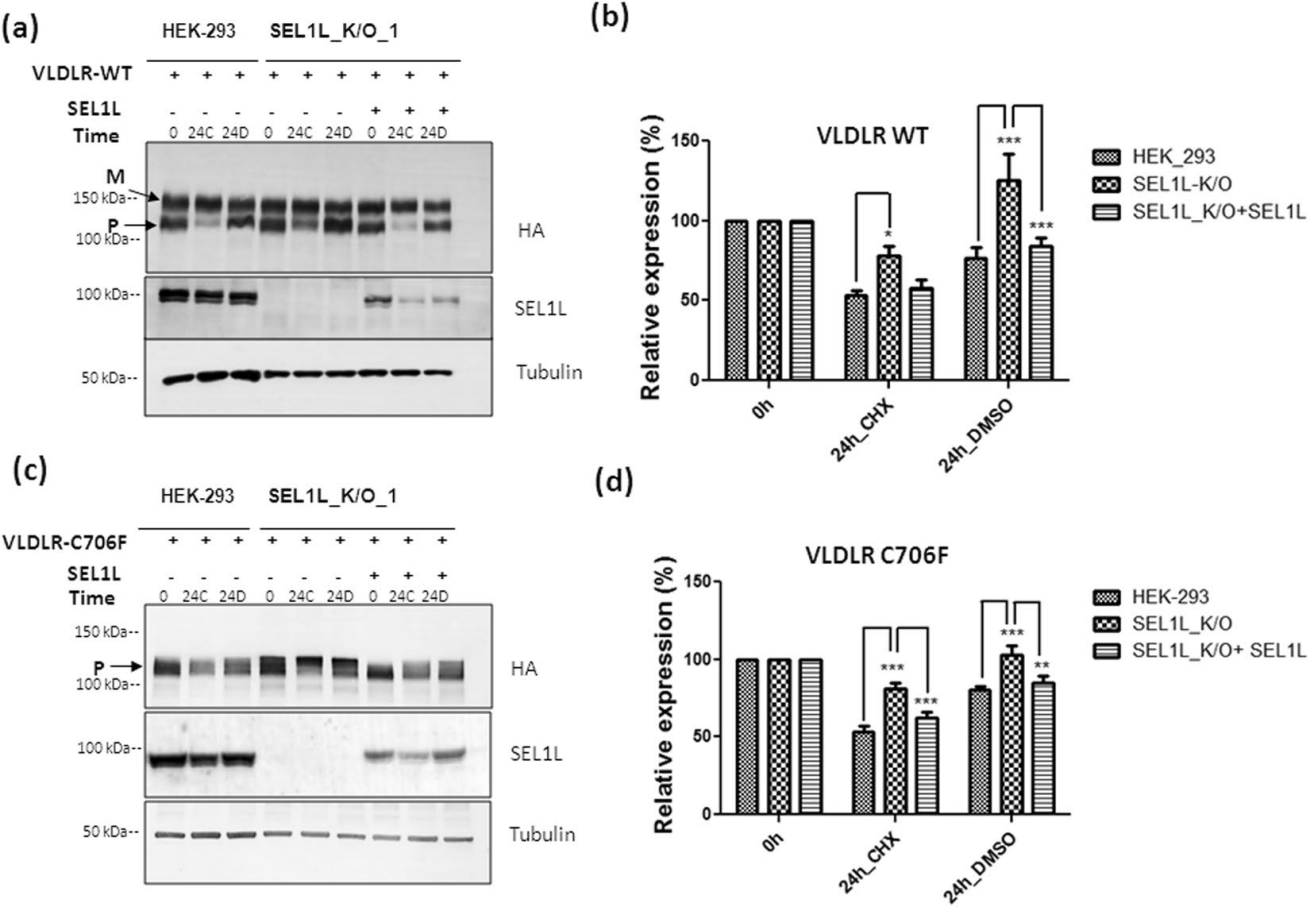
Exogenous expression of SEL1L enhances the degradation of VLDLR WT and mutant C706F in SEL1L Knockout cell lines: (**a**) HEK-293 and SEL1L K/O cells were transfected with VLDLR-WT plasmid alone or co-transfected with VLDLR-WT and SEL1L constructs. At 24 h post-transfection, the cells were treated with 100 µg/ml CHX (24 C) or DMSO (24D) for 24 h and cells were harvested for western blot analysis. Total cell lysates were analysed by immunoblotting against antibodies for HA, tubulin and SEL1L. **(b)** Graph representing densitometric analysis of 6 independent experiments conducted in knock-out cells generated by different gRNAs. (*) *p* ≤ 0.05; (***) *p* ≤ 0.001; Two way ANOVA. **(c)** Cells expressing VLDLR mutant C706F alone or SEL1L as described in (a) were treated with CHX for 24 h and western blots were generated as in (**a**). **(d)** Densitometric analysis of six independent experiments from knock-out cell lines generated by different gRNAs, (*) *p* ≤ 0.05; (**); *p* ≤ 0.01; (***) *p* ≤ 0.001; Two way ANOVA, Bonferroni post-test. Regions cropped from separate images are demarcated with borders. Unprocessed original scans of western blots are shown in Supplementary Figure S13.

We next examined the effect of exogenous SEL1L overexpression on the stability of VLDLR in the knock-out cell lines. VLDLR wild type and C706F mutant were transiently co-transfected with SEL1L expression plasmid in HEK-293 or knock-out cells. The exogenously expressed level of SEL1L was lower than that of endogenous expression levels in HEK-293 cells (Fig. 7). The cells were treated with cycloheximide or DMSO for 24 h to assess the degradation of VLDLR WT and mutant. When SEL1L was co-transfected, VLDLR WT half-life was observed to be declined in the presence of cycloheximide (Fig. 7a & b). Similarly, the C706F mutant degradation was found to be enhanced when SEL1L was overexpressed in knock-out cells (Fig. 7c & d). The results were reproducible in different knockout cell lines generated by different gRNAs targeting *SEL1L* gene (Supplementary Figures S6 and S7). Taken together our results suggest that SEL1L is involved in the ER quality control of VLDLR WT and mutants.

## Discussion

We have analysed the degradation routes of three missense mutants of VLDLR (D487Y, D521H and C706F) implicated in the autosomal recessive disorder DES. Our results indicated that the VLDLR mutants were retained in the ER for a prolonged period of time and were not rapidly degraded as other classical ERAD substrates. The most common disease associated variant of the cystic fibrosis transmembrane conductance regulator(CFTR), CFTRΔF508, is highly unstable and is rapidly degraded by the ubiquitin-proteasomal system^30^. While pathogenic alpha-1 antitrypsin (AAT) mutant AAT-Z, though one of the model substrates of ERAD pathway, has been reported to accumulate in the ER as insoluble aggregates and cleared away by autophagy^30^. To explore the biochemical characteristics of the VLDLR mutants that render them resistant to degradation, we studied the aggregation status and interaction of the mutant protein with ER quality control components. Solubility assay indicated that the mutant VLDLRs were more prone to aggregation than the wild type and elicit ER stress in cells overexpressing them as indicated by induction of spliced XBP-1s mRNA. Misfolded proteins that are retained in the ER without undergoing degradation have been reported to cause ER stress and activate UPR pathways^31^. When we examined the degradation routes of VLDLR mutants, the degradation of all three mutants was found to be sensitive to inhibitors of both proteasome and lysosome. Our results suggest that proteasome inhibition had a pronounced effect on the accumulation of ubiquitinated species of mutants when compared to lysosomal inhibition. Although the ubiquitin-proteasome system and macroautophagy have been deemed to be separate entities, a growing body of evidence indicate that the two systems cross-talk since ubiquitinated proteins are targeted for degradation via both pathways^32^.

In the ER, newly synthesized polypeptides are allowed ample opportunities to fold prior to proceeding to ERAD. The lectin chaperones in the ER, calnexin and calreticulin play crucial roles in assisting folding and preventing aggregation and retaining immature or misfolded cargo in the ER^33^. During this phase, *N*-glycosylated ERAD candidates remain trapped in the calnexin/calreticulin chaperone system until they meet the ERQC standards for export. Extended interaction with calnexin has been reported to be responsible for the ER-retention of mutants in several diseases^34–36^. The VLDLR WT and all the three missense mutant proteins were observed to interact with calnexin in transfected HEK-293T cells. Even though a significant amount of the precursor form was present in the immunoprecipitates of the wild type receptor, a very faint signal of calnexin was detected in immunoblots, indicating that the folding-competent forms of wild type do not form stable interactions with calnexin. On the other hand, all the three mutants showed strong association with calnexin, suggesting a role for calnexin in the ER retention of VLDLR mutants. Mislocalized mutant proteins trapped in ER has been demonstrated to be diverted to the ERAD pathway^37^ or rescued to the cell surface by reducing the interactions of client proteins with castanospermine, a glucosidase inhibitor^38,39^.

The VLDLR mutants and to a lesser extent wild type were found to interact with SEL1L, an adaptor protein for the E3 ligase HRD1. In mammals, HRD1/SEL1 complex has been considered to participate in the ERAD of secreted proteins with misfolded lumenal domains^26^. SEL1L plays a crucial role as a linker that recruits ERAD substrates through association with ER-resident lectins/chaperones^29,40,41^. Down-regulation of SEL1L has been reported to block degradation of various ERAD substrates including truncated ribophorin (RI332)^40^, the null Hong Kong (NHK) variant of α1-proteinase inhibitor (α1-antitrypsin)^29^. However, even though limited in number, recent studies indicate that the mammalian ERAD does not seem to follow rigid rules for substrate recognition since some endogenous transmembrane proteins have also been found to be substrates of HRD1/SEL1L complex^27,28,42^. Recently it was reported that HRD3, the yeast homologue of SEL1L, has a direct role in ERAD-M, since removal of HRD3 alone stabilized the degradation of some ERAD-M substrates in yeast^43^. The VLDLR mutant C706F is an example of a misfolded transmembrane protein with lumenal lesion.

By co-IP and CRISPR-Cas9 mediated functional knockout of SEL1L, we have shown that SEL1L plays a crucial role in the degradation of misfolded VLDLR mutant C706F. Further studies are required to unveil whether SEL1L associated degradation of VLDLR mutants involve HRD1 mediated ERAD, though a growing body of literature suggests that SEL1L has direct and essential functions in ERAD, independent of HRD1^43^. VLDLR wild type was also found to interact weakly with SEL1L and the degradation of the wild type was found to be stabilized to an extent in SEL1L knockout cell lines. Though the wild type VLDLR has been reported to be degraded through the lysosomal pathway, our results have indicated the accumulation of VLDLR WT in HEK-293 cells, in response to proteasome inhibition. SEL1L has been reported to be critical for the stability of HRD1 and SEL1L/HRD1 complex^44^. In SEL1L knockout cell lines, HRD1 levels were found to be strikingly reduced(Supplementary figure S4a). Therefore general suppression of ERAD activity could also have contributed to stabilizing effects observed for both VLDLR WT and C706F mutant. Intriguingly, in a recent study where genome-wide microarray analysis of colonic epithelium was conducted in an enterocyte-specific Sel1L-knockout mice, the topmost down-regulated pathway was the lysosomal pathway^42^.

It would be worthwhile to explore the possibility of natural interactions between VLDLR and SEL1L in different cell types, since SEL1L is emerging to have diverse roles in ER homeostasis and lipid metabolism^45,46^. Recently it was reported that SEL1L plays an ERAD independent role in the maturation and processing of Lipoprotein Lipase (LPL) and systemic lipid metabolism^46^. It is noteworthy that VLDLR works in concert with LPL in many tissues and induce receptor-mediated lipoprotein catabolism^47^. Deficiency of SEL1L in adipocytes leads to ER-retention and aggregation of LPL, which are degraded primarily by autophagy. Mice with adipocyte-specific Sel1L deficiency are resistant to diet-induced obesity and exhibit postprandial hypertriglyceridemia^46^. Mice lacking the VLDLR receptor and 50% of the DES patients were reported to be protected from obesity^48^. Further, a homozygous missense mutation in SEL1L gene was reported to be associated with a canine progressive cerebellar ataxia.^49^. Further studies in this direction are needed to elucidate the exact relationship between VLDLR and SEL1L.

In conclusion we report here that the ER-retained VLDLR mutants implicated in DES are aggregation-prone, cause ER stress and degraded predominantly by the proteasomal pathway. We also demonstrate the stable interactions of the mutants with calnexin and ERAD adaptor protein SEL1L. Though further investigations are required to extend the conclusions of this study to the *in vivo* effects of the mutation, our studies provide insight into the intrinsic properties of the mutants and their interaction with ERQC, which will help to devise strategies for reduction of aggregation or enhance the degradation in relevant scenarios.

## Methods

### Antibodies

The antibodies with their dilutions and sources were as follows: Antibodies for western blotting: rabbit polyclonal anti-HA (1:4000; H6908, Sigma, Lot No: 022M4806), mouse monoclonal anti-α-tubulin (1:10,000; Sigma, T5168, Lot No: 103M4773V), goat anti-SEL1L (1: 200; Santa Cruz Biotechnology, SC-48081, Lot No: C3109), Rabbit anti-HRD1 (1:500: Cell Signaling technology, 12925 S, Lot No: 1), rabbit anti-OS-9 (1: 500: Abcam, ab19853, Lot No: GR54041-1), rabbit anti-Calnexin (1:1000; Cell Signaling Technology, 2433 S, Lot No: 2), mouse monoclonal anti-ubiquitin (1:1000; Sigma, U0508, Lot No: SLBL1928V), Rabbit anti-Histone-H3 (1:1000; Cell Signaling Technology, 9715S, Lot No: 18), Rabbit anti-GAPDH (1: 2500; Abcam, ab9485, Lot No: GR184357-1), Rabbit anti-LC3-B (1: 1000; Sigma, L7543, Lot No: 046M4787V), goat anti-rabbit IgG-peroxidase (1: 50,000; Sigma), rabbit anti-mouse IgG-peroxidase (1:80,000; Sigma), chicken anti-goat IgG-peroxidase (1:5000, Santa Cruz Biotechnology).

### Cell culture, transfection and treatments

Human embryonic kidney cells (HEK-293, HEK-293T, ATCC) were cultured in Dulbecco’s modified Eagle’s medium/F12 medium (Invitrogen) supplemented with 10% fetal bovine serum (Invitrogen), penicillin (10 U/ml) and streptomycin (100 μg/ml) at 37 °C with 5% CO_2_. For transfection, cells were grown in 6-well tissue culture plates and transfected with 1 μg plasmid DNA using FuGENE HD transfection reagent (Promega).

For translation arrest, 24 h after transfection, cells were cultured in serum-free medium for 8-16 hours and incubated with cycloheximide (100 µg/ml) for various time periods. For proteasome blocking, serum-starved cells were cultured in the presence of MG132 (10 µM), ALLN (10 µM), Lactacystin (10 µM) prior to adding cycloheximide. For blocking lysosomal degradation, Leupeptin (0.1 mM) and NH_4_Cl (20 mM) were added to the culture medium. Cells were harvested for protein extraction at different time intervals.

### Immunoprecipitation and Western blotting analysis

Forty eight hours after transfection, HEK-293T cells were lysed in IP lysis buffer (Pierce Inc.) containing protease inhibitors (SigmaFAST protease inhibitor cocktail, Sigma) according to the manufacturer’s instructions. Total protein concentration was determined by Bicinchoninic Acid protein Assay (BCA kit, Pierce). HA-tagged proteins were immunoprecipitated using anti-HA agarose beads (Pierce). Briefly, Equal amounts of total cell lysates were incubated with anti-HA agarose beads for 2 h at 4 °C with rotation. Immunoprecipitates were collected by centrifugation and washed thrice with lysis buffer. For Western blotting, the proteins were eluted from the beads by boiling in Laemmli sample buffer. The samples were then resolved on 7.5% SDS-PAGE gel or precast 4–20% gradient gels (Bio-Rad) followed by blotting onto nitrocellulose membranes (Whatman Protran) or PVDF (Thermo Fisher Scientific) and probed with respective antibodies. Detection was performed using Enhanced Chemiluminescence Plus reagent (Pierce) and Typhoon FLA 9500 Imager (GE Healthcare Biosciences). Densitometric analysis of the blots was performed by Image Studio Lite (Li-COR Biosciences) software and statistical analysis and representations were generated by GraphPad Prism software. Triton X-100 solubility assay was carried out as described in^50^. For analyzing ubiquitylation, cell extracts were prepared in RIPA buffer containing protease inhibitors and N-Ethylmaleimide (NEM). For detecting ubiquitinylated VLDLR the nitrocellulose membranes were boiled in deionized water after transfer before the blocking step in western blotting.

### CRISPR/Cas9 mediated *SEL1L* knock-out in HEK-293 cells

The GeneArt® CRISPR Nuclease Vector with orange fluorescent protein (OFP) Reporter (Life Technologies) system was used for the CRISPR/Cas9 genome editing in mammalian cells according to the protocol provided by the manufacturer. Two pairs of guide RNAs were designed for editing exon3 of the *SEL1L* gene. gRNA1_FWD: GTA AAG GAC CAT ACT ACT GCG TTT T, gRNA1_REV: GCA GTA GTA TGG TCC TTT ACC GGT G, gRNA2_FWD: ACT GCA GGC AGA GTA GTT GCG TTT T, gRNA2_REV: GCA ACT ACT CTG CCT GCA GTC GGT G. The plasmids were sequenced to confirm the cloning of the gRNAs in the correct orientation. The OFP/Cas9 plasmids harboring the gRNAs were transfected into HEK-293 cells using Fugene HD reagent. Forty eight hours after transfection, Cas9 and CRISPR RNA expressing cell populations were enriched using fluorescence-activated cell sorting (FACS). Single clones were isolated by limiting dilution seeding of FACS enriched OFP positive cell pool (BD FACSCanto II, BD Biosciences). For screening for positive clones, cells were lysed in direct PCR lysis buffer and the lysates were used directly for amplifying the targeted region in the genomic DNA using primers flanking the target site. Gene-editing was analysed by sensitivity to restriction enzymes adjacent to the PAM sites. The introduction of indels was confirmed by sequencing and gene knockout was validated by immunoblotting analysis of the SEL1L protein levels.

### Quantitative real-time PCR

Following transfection, total RNA was isolated from the transfected cells using Promega SV total RNA isolation system. Total RNA was subjected to reverse transcription using Promega GoScript reverse transcriptase kit by following the manufacturer’s instructions. Quantitative real-time PCR was performed on a QuantStudio 7 Flex (Applied Biosystems) real-time PCR machine. TaqMan real-time PCR assays (Life Technologies) for XBP-1s (Hs03929085_g1) was used for analyzing ER stress and as an internal reference control GAPDH (Hs02758991_g1) was used, according to the manufacturer’s protocol. Gene expression was analyzed by comparative Ct (∆∆Ct) method using QuantStudio Real-Time PCR software v1.2. Statistical analysis was performed by one-way ANOVA followed by Bonferroni *post hoc* test using GraphPad Prism software.

### Cloning of *SEL1L* gene

*SEL1L* ORF (RefSeq accession: NM_005065.5) was amplified from cDNA prepared from total RNA from HEK-293 cells using the following primers; SEL1L_Hind FP: AGA CGC AAG CTT TGG CAG AGG CGA AGG CGA C, SEL1L _Bam_FP: ACT AGT GGA TCC TTA CTG TGG TGG CTG CTG CTC. The amplified PCR product was cloned into the HindIII/BamH1 sites of *pcDNA 3.0* vector and sequenced completely by Sanger sequencing. The *SEL1L* gene cloned in *pcDNA* was found to be unstable and for plasmid propagation *E. coli* strain Stbl3 was used at 30 °C.

### Statistical analysis

Statistical analysis between two groups was conducted by two-tailed unpaired Student's *t*-test. For comparison of the time-dependent protein degradation between mutants or more than two groups, two-way ANOVA tests (difference between two groups of points) were performed followed by Bonferroni *post hoc* tests (difference between the two points at each given time point) in GraphPad Prism software. Significance was established at *P* < 0.05. In all graphs, error bars indicate SEM, and the biological replicate numbers are indicated as the **n** numbers in the legends.

### Data availability

All data generated or analyzed during this study are included in this published article (and its Supplementary Information files).

## Electronic supplementary material

Supplementary Files
