# Supplementary material for: Degradation routes of trafficking-defective VLDLR mutants associated with Dysequilibrium syndrome
Source: Sci Rep. 2018 Jan 25;8:1583. doi: 10.1038/s41598-017-19053-8 (PMC5785505; doi:10.1038/s41598-017-19053-8)
Supplement: Supplementary file 1 — Supplementary Files [file 41598_2017_19053_MOESM1_ESM.pdf]

Degradation routes of trafficking-defective VLDLR mutants associated with Dysequilibrium syndrome

Praseetha Kizhakkedath[1], Anne John[1], Lihadh Al-Gazali[2], Bassam R. Ali[1,3]

[1]Department of Pathology, College of Medicine and Health Sciences, United Arab Emirates University, Al-Ain, United Arab Emirates. [2]Department of Paediatrics, College of Medicine and Health Sciences, United Arab Emirates University, Al-Ain, United Arab Emirates. [3]Zayed Center for Health Sciences, United Arab Emirates University, Al-Ain, United Arab Emirates.

Supplementary Figures

Figure S1

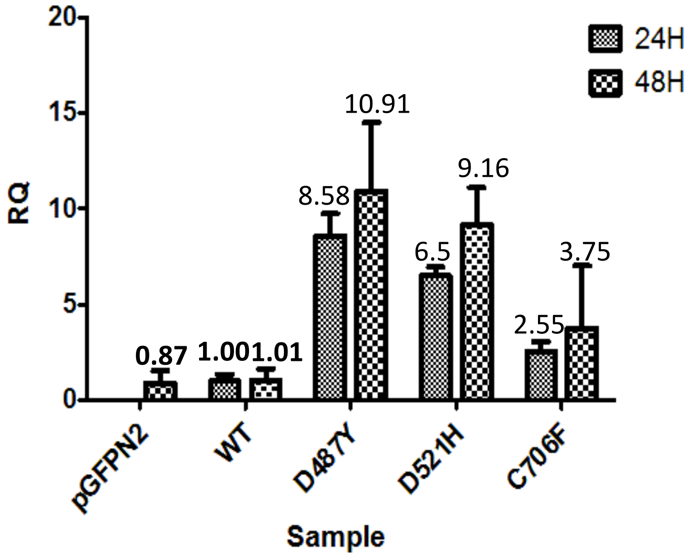

Figure S1 : Time-dependent increase in the level of XBP-1s in HEK-293T cells expressing VLDLR mutants. Aggregation-prone VLDLR mutants induce ER stress, represented through elevated alternatively spliced XBP-1 transcript levels, measured through quantitative PCR (qPCR). The XBP-1s mRNA levels of the VLDLR WT at 24h post-transfection was set as 1.00. Fold-changes in mRNA expression of the mutants were expressed in relation to WT. Error bars represent ± S.E.M. of three experiments

Figure S2

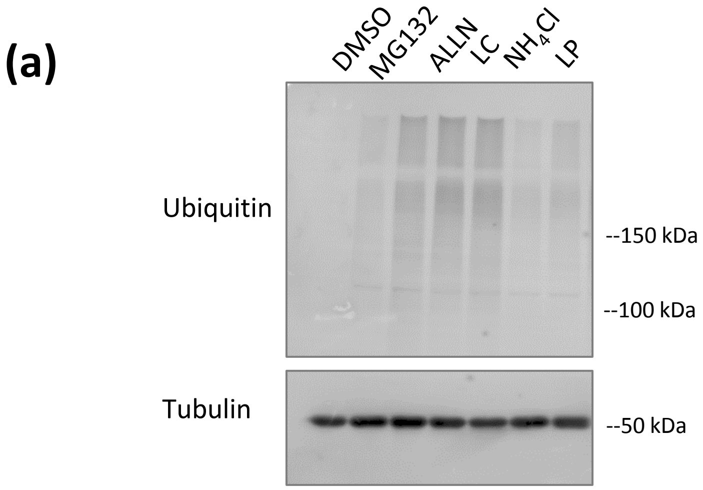

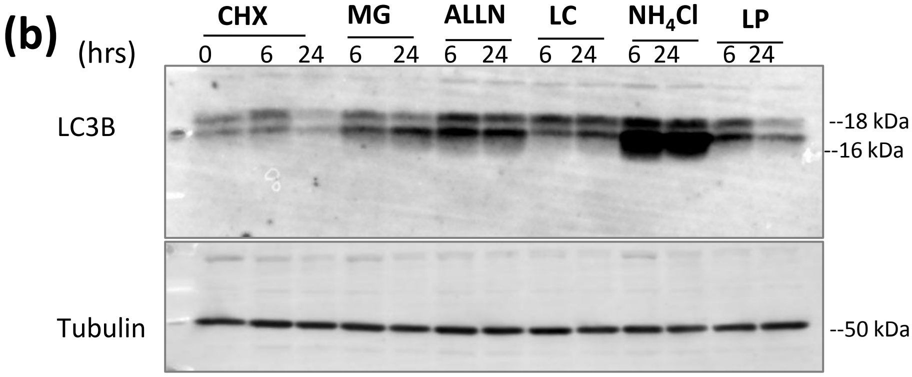

Figure S2 : (a) Accumulation of total ubiquitylated proteins in HEK-293 cells treated with DMSO(-) or either proteosome inhibitors (10µM MG132, 10µM ALLN or 10µM lactacystin (LC)) or lysosome inhibitors (0.1mM leupeptin (LP) or 20mM NH_4Cl) for 16h. Tubulin was used as loading control. **(b)** Western blot showing accumulation of the autophagy reporter LC3B as a positive control for lysosomal inhibition. HEK-293 cells transiently expressing the VLDLR -C706F mutant was treated with cycloheximide (CHX) in the presence of proteosome inhibitors (10µM MG132, 10µM ALLN or 10µM lactacystin) or lysosome inhibitors (0.1mM leupeptin or 20mM NH_4Cl) as indicated. Total cell lysates were probed with anti-LC3B antibody.

Figure S3

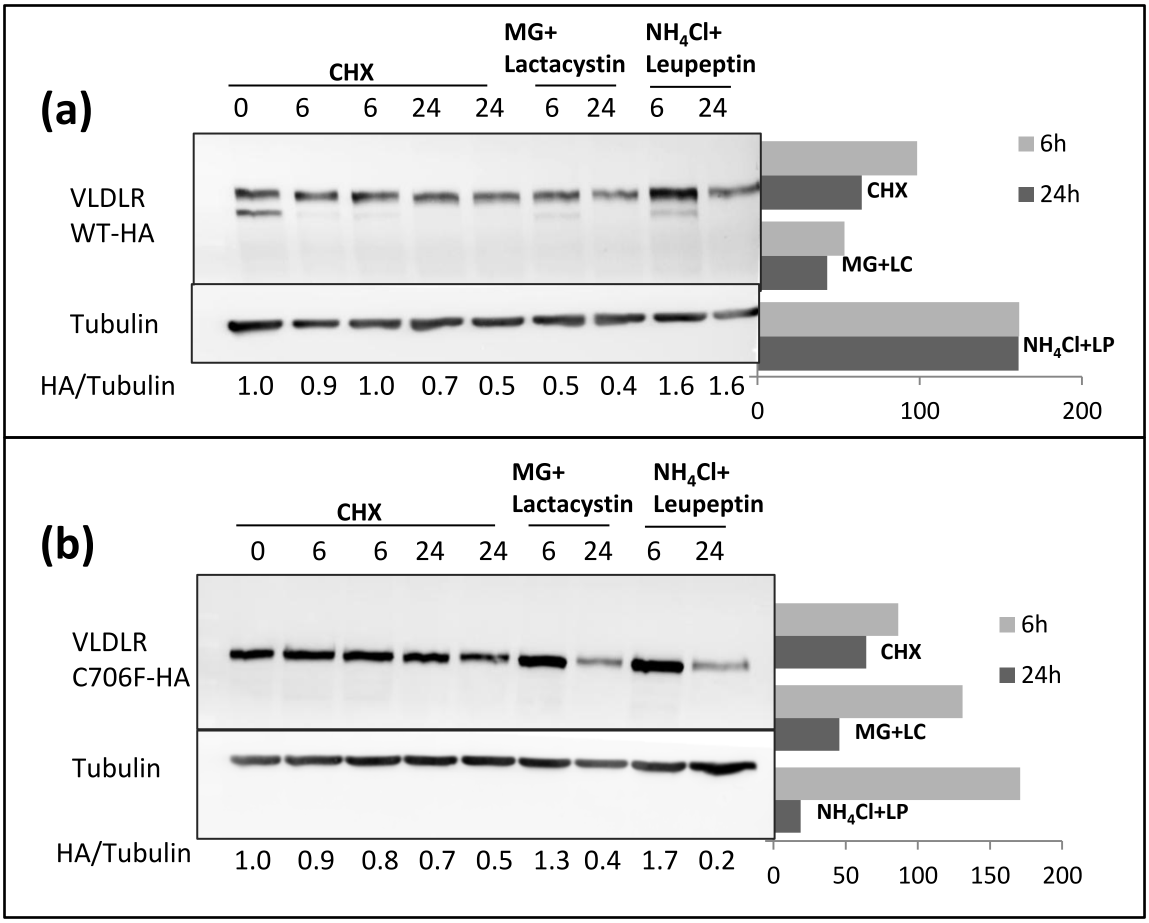

Figure S3: Representative figure showing the effect of combinations of proteasomal or lysosomal inhibitors on the degradation rates of VLDLR WT and mutants. HEK-293 cells transiently expressing the *VLDLR WT* **(a)** or *C706F* mutant **(b)** were treated with 150µg/µl cycloheximide (CHX) in the presence of a combination of inhibitors of proteasome (10µM MG132(MG)+ 10µM lactacystin (LC)) or lysosome (0.1mM leupeptin (LP)+ 20mM NH_4Cl) as indicated. Total cell lysates were analyzed by immunoblotting against HA. Relative amounts of respective proteins remaining at the indicated time points were quantified and normalized to tubulin levels. Tubulin-normalized VLDLR protein levels at 0h were defined as 1.0 for each panel.

Figure S4

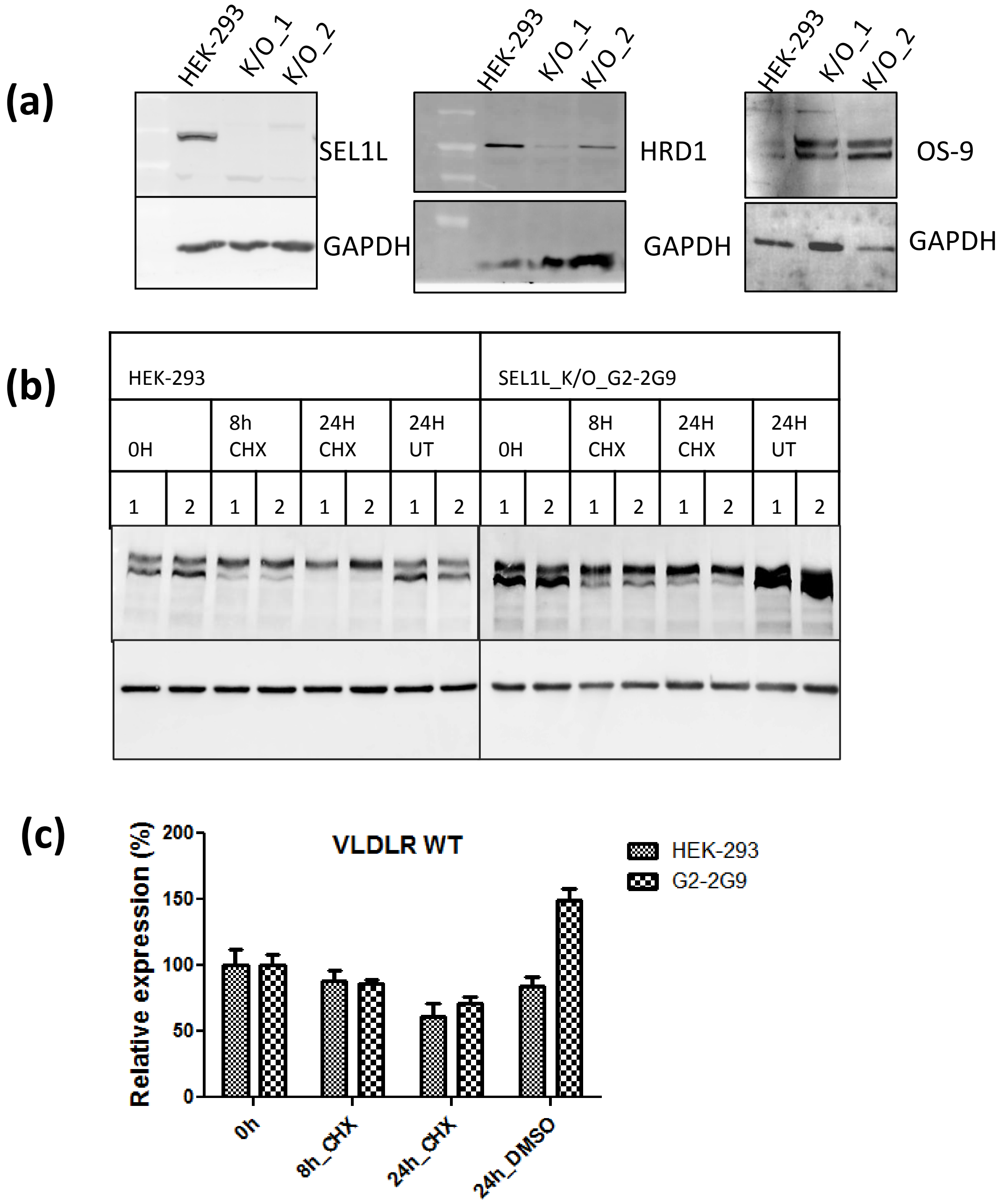

Figure S4: (a) Analysis of expression levels of SEL1L, HRD1 and OS-9 in SEL1L knockout (K/O) cell lines. **(b)** Delayed degradation of VLDLR WT in SEL1L knockout cell line generated by gRNA2 (g2-2G9). VLDLR WT was transfected into HEK-293 cell lines and SEL1L knockout cell lines (SEL1L_K/O_g2-2G9) in parallel. At 24h after transfection, the cells were incubated with 100 µg/ml CHX for 24h and collected at different times (8h & 24h) for western blot. Replicates for each time point were analyzed in the same blot. The blots were probed against HA and tubulin. Tubulin-normalized VLDLR protein levels at 0h were defined as 100%. **(c)** Densitometric analysis of two experiments

Figure S5

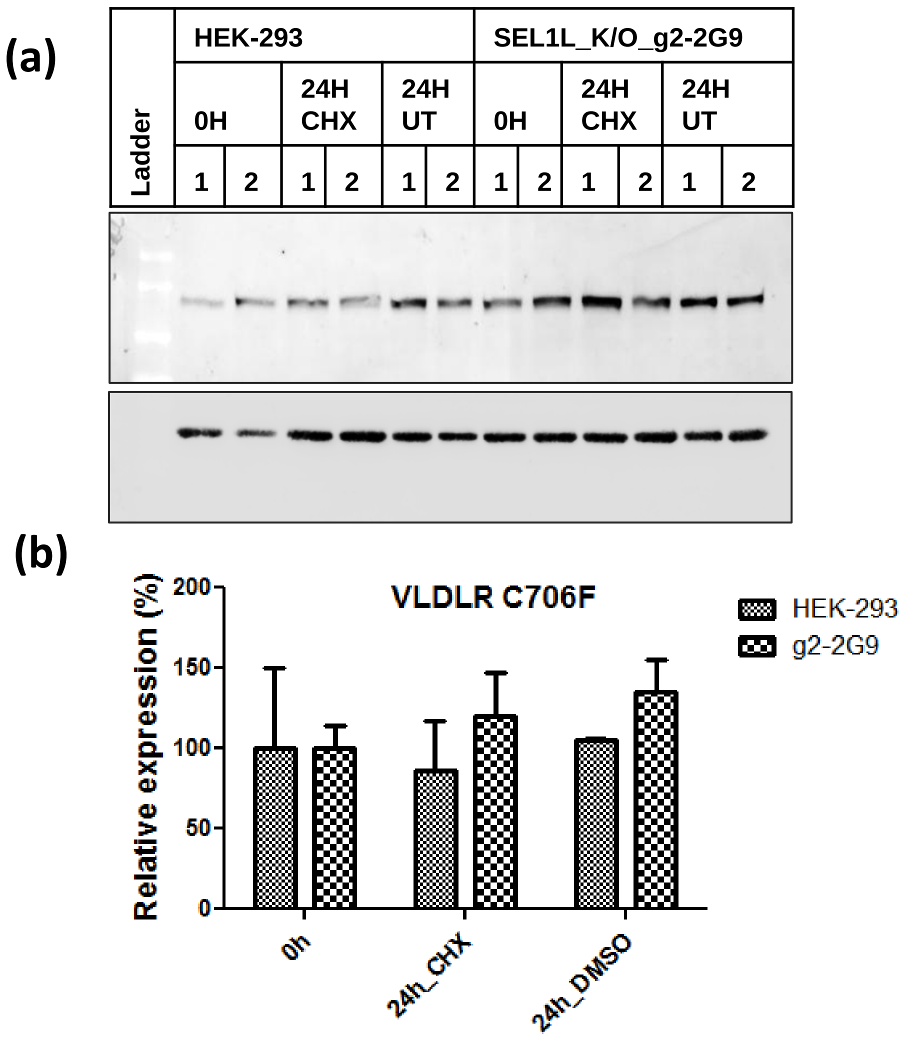

Figure S5: Analysis of degradation of VLDLR mutant C706F in SEL1L knockout cell line generated by gRNA2 (SEL1L_K/O_g2-2G9). VLDLR C706F was transfected into HEK-293 cell lines and SEL1L_K/O_g2-2G9 cell lines in parallel. At 24h after transfection, the cells were incubated with 100 µg/ml CHX for 24 h and collected at 24h for western blot. Replicates for each time point were analyzed in the same blot. The blots were probed against HA and tubulin. Tubulin-normalized VLDLR protein levels at 0h were defined as 100%. **(a)** Immunoblots showing the turn-over of VLDLR-WT protein. **(b)** Densitometric analysis of the two independent experiments

Figure S6

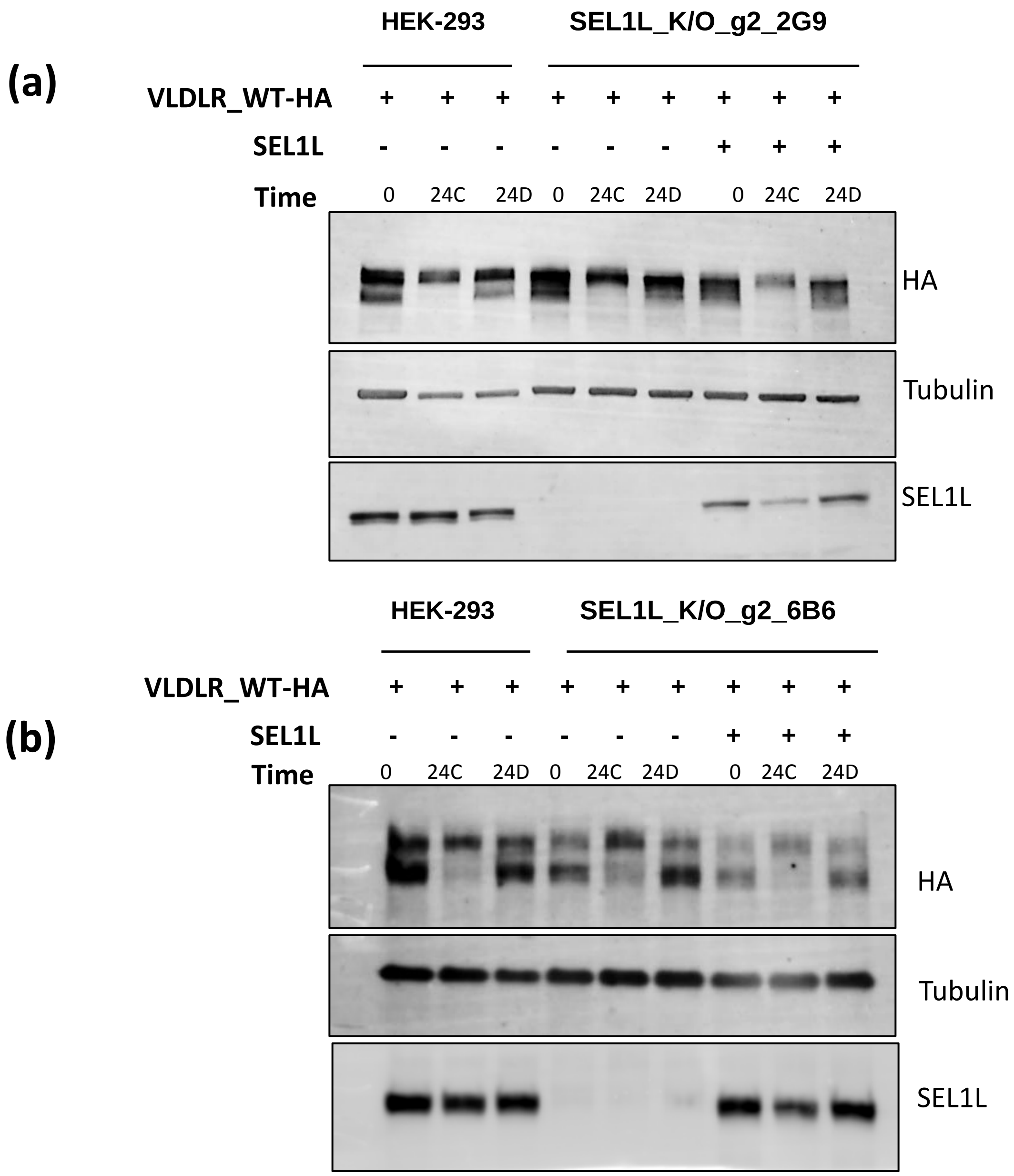

Figure S6: Exogenous expression of SEL1L enhances the degradation of VLDLR WT in different SEL1L knockout cell lines **(a)** SEL1L_K/O_g2-2G9: HEK-293 and SEL1L_K/O_g2-2G9 cells were transfected with *VLDLR-WT* plasmid alone or co-transfected with *VLDLR-WT* and *SEL1L* constructs. At 24h post-transfection, the cells were treated with 100 µg/ml CHX (24C) or DMSO(24D) for 24h and cells were harvested for western blot analysis. Total cell lysates were analysed by immunoblotting against antibodies for HA, tubulin and SEL1L. Tubulin-normalized VLDLR protein levels at 0h were defined as 1.0. **(b)** Similar experiment as in **(a)** in a different knockout clone, SEL1L_K/O_g2-6B6

Figure S7

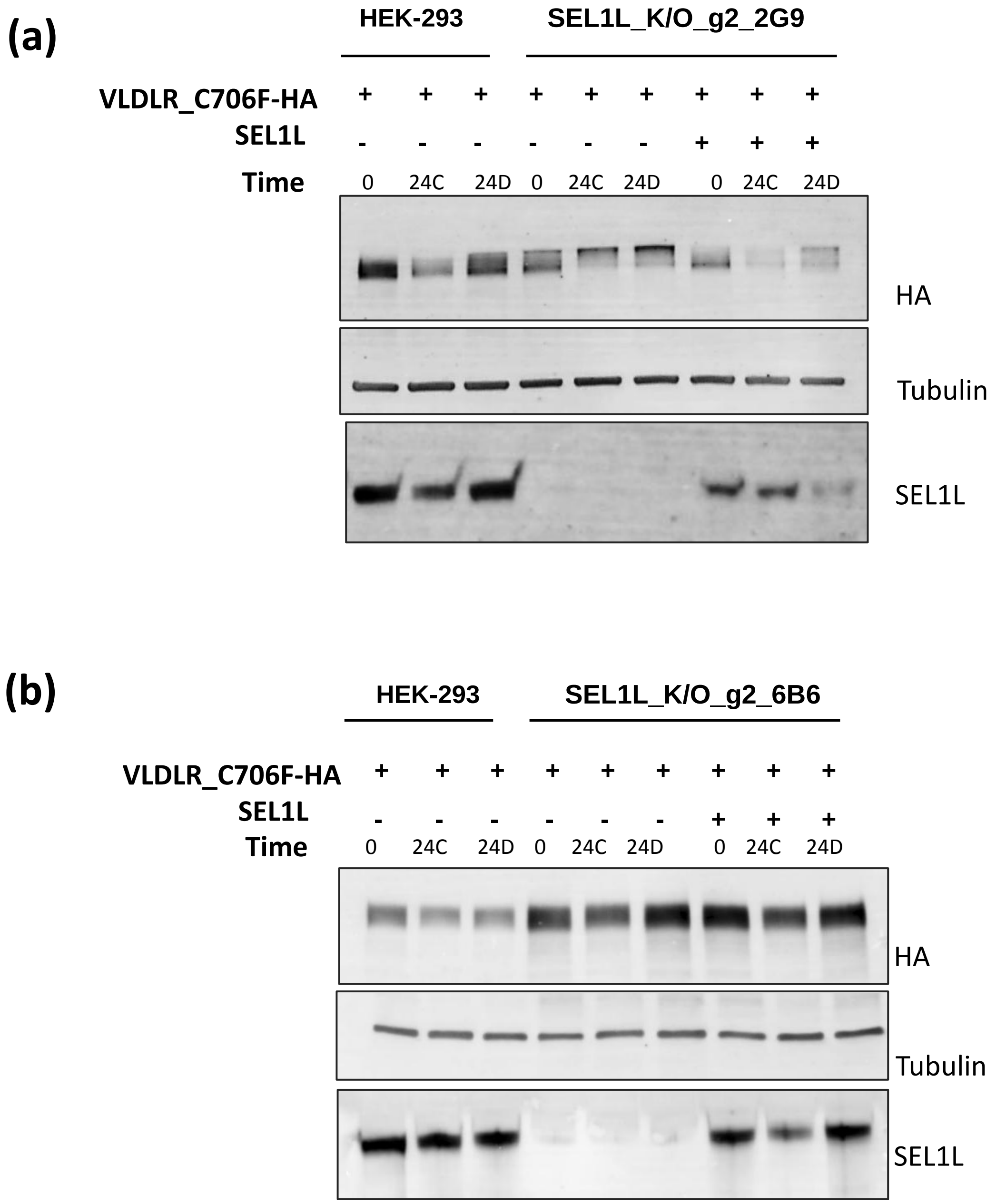

Figure S7: Exogenous expression of SEL1L enhances the degradation of VLDLR-C706 in different SEL1L Knockout cell lines: (**a**)HEK-293 and SEL1L_K/O_g2-2G9 cells were transfected with *VLDLR-C706F* plasmid alone or co-transfected with *VLDLR-C706F* and *SEL1L* constructs. At 24h post-transfection, the cells were treated with 100 µg/ml CHX (24C) or DMSO(24D) for 24h and cells were harvested for western blot analysis. Total cell lysates were analysed by immunoblotting against antibodies for HA, tubulin and SEL1L. Tubulin-normalized VLDLR protein levels at 0 h were defined as 1.0. **(b)** Similar experiment as in **(a)** in a different knockout clone, SEL1L_K/O_g2-6B6

Figure S8

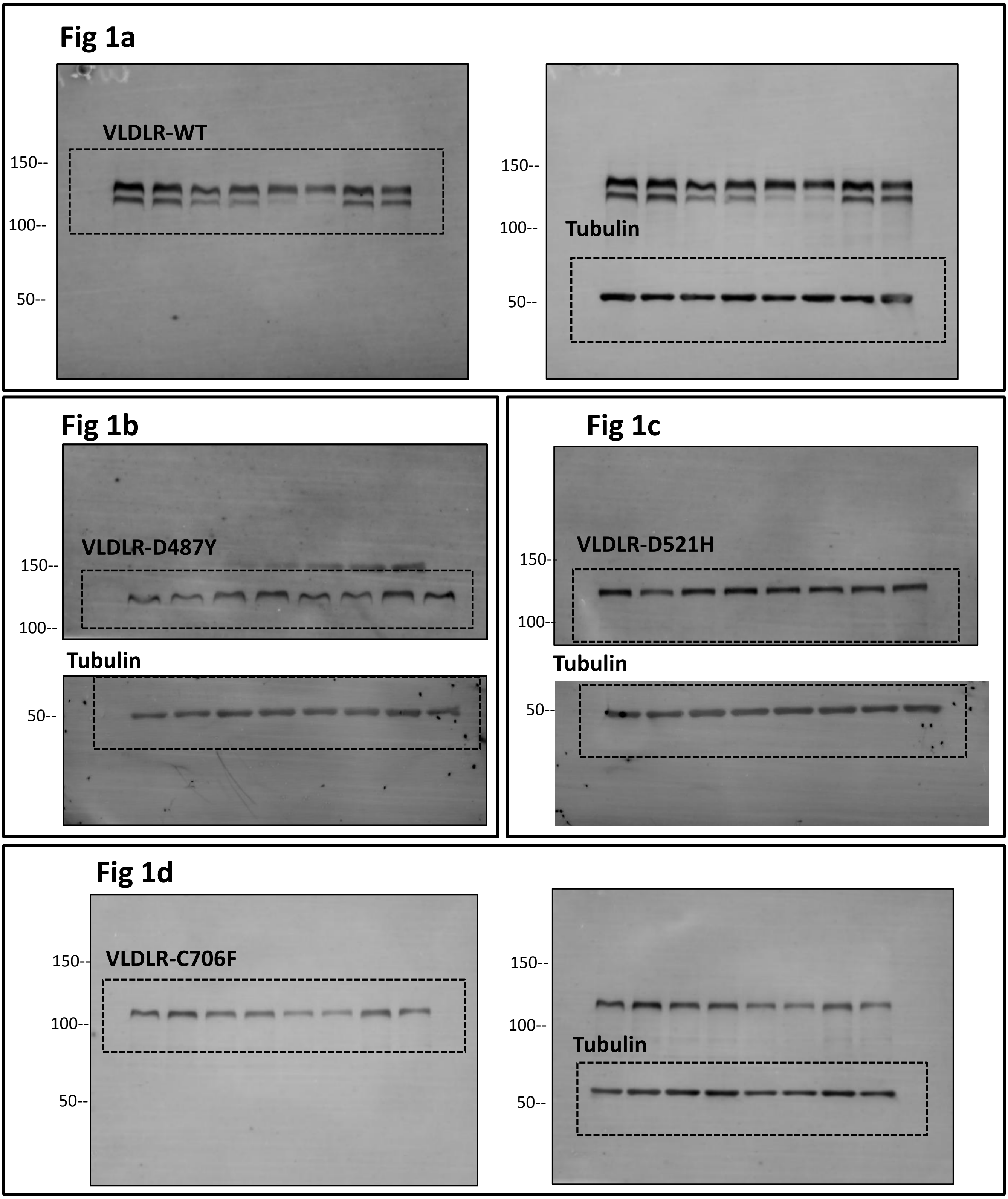

Figure S8 : Original scans of western blots displayed on Fig 1a, b, c and d, with the cropped regions marked.

Figure S9

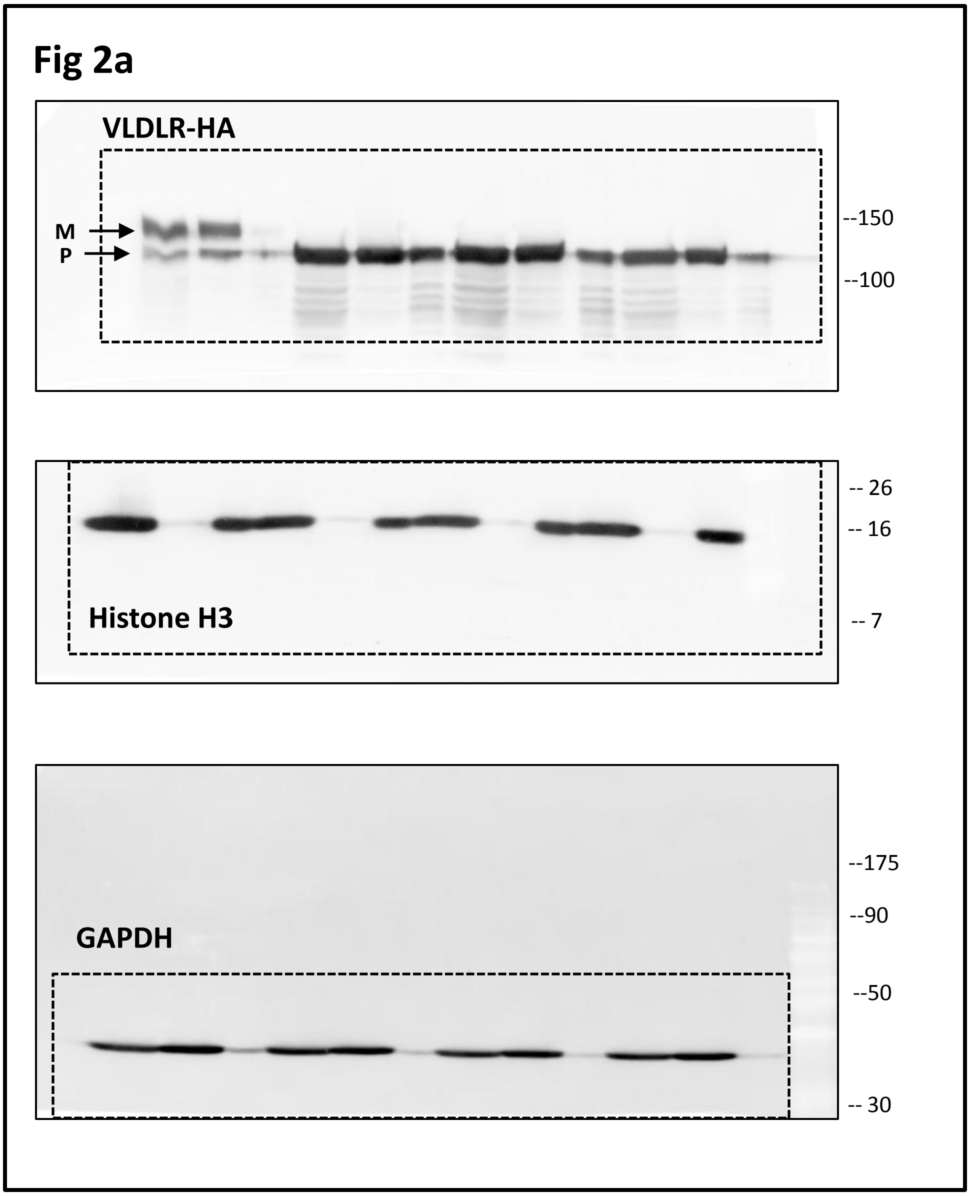

Figure S9 : Original scans of western blots displayed on Fig 2

Figure S10

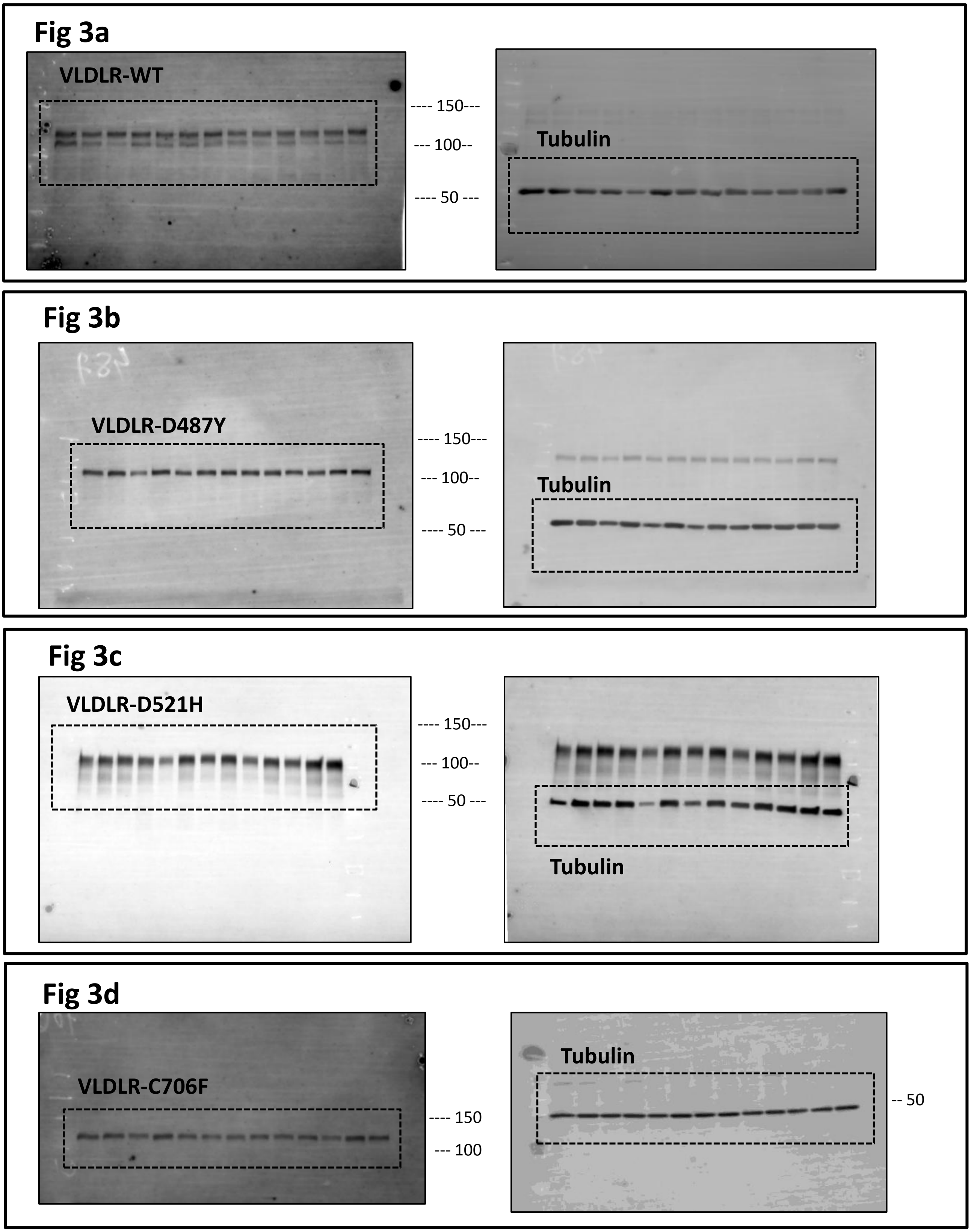

Figure S10 : Original scans of western blots displayed on Fig 3a, b, c and d

Figure S11

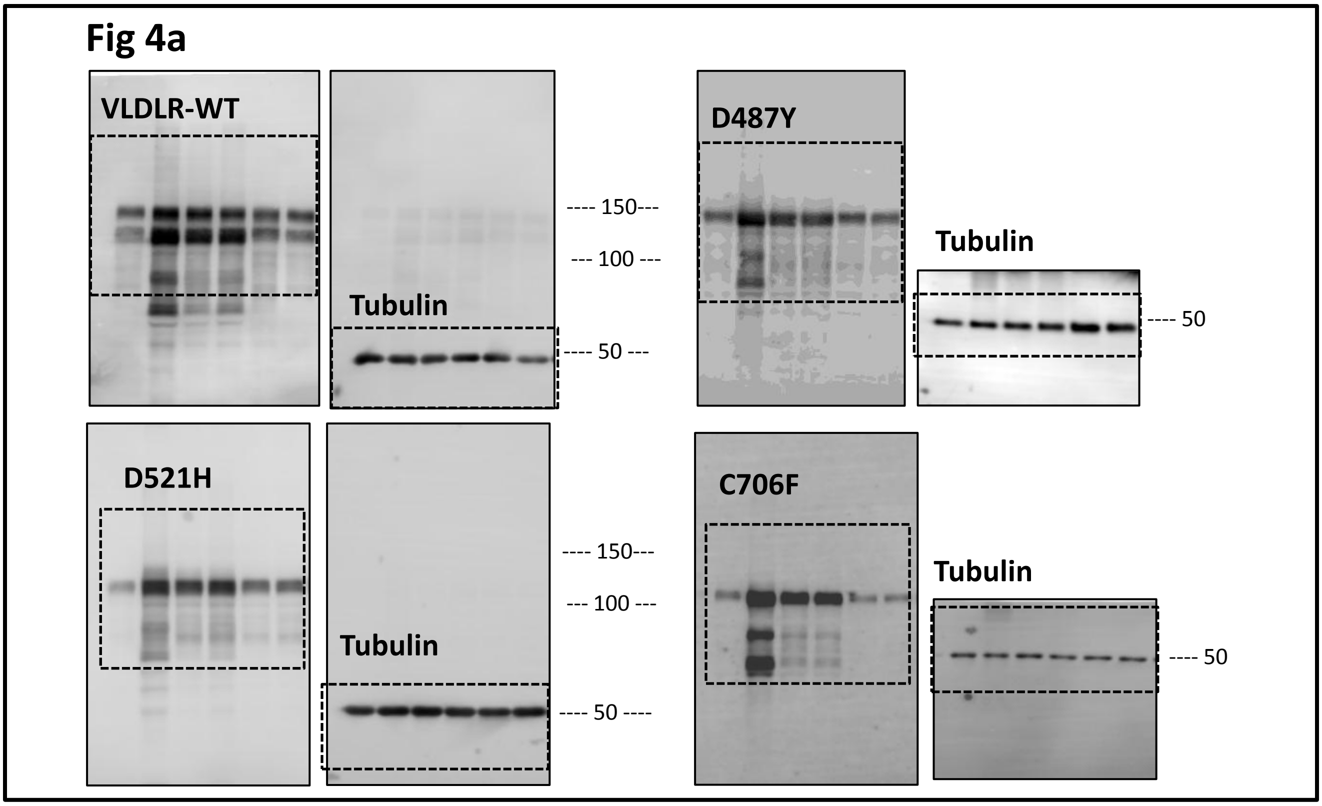

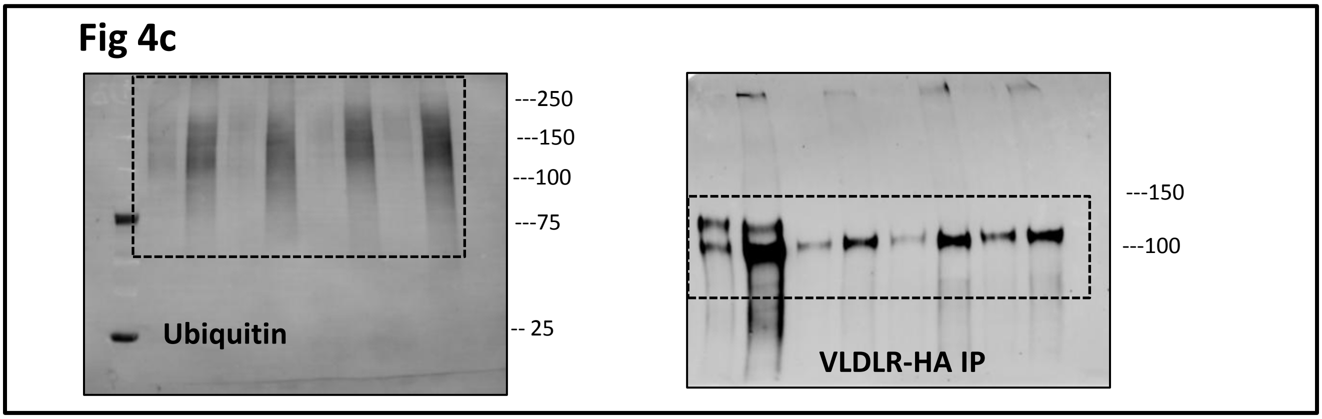

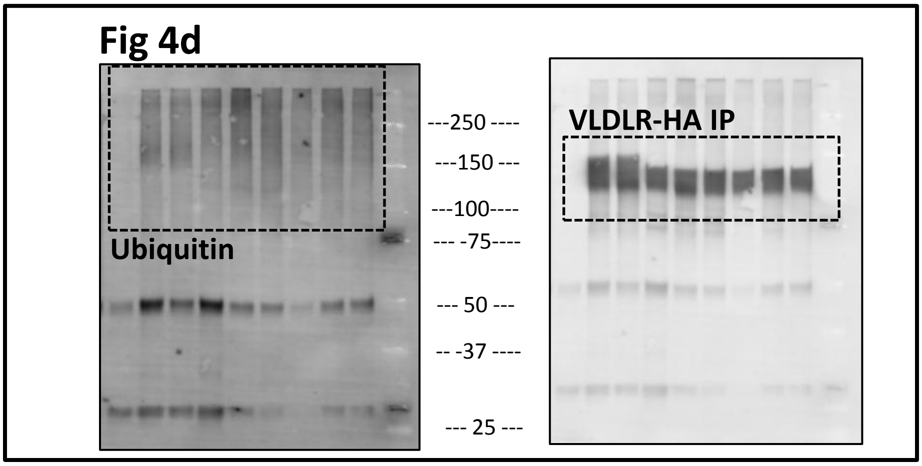

Figure S11: Original scans of western blots displayed on Fig 4a, c and d

Figure S12

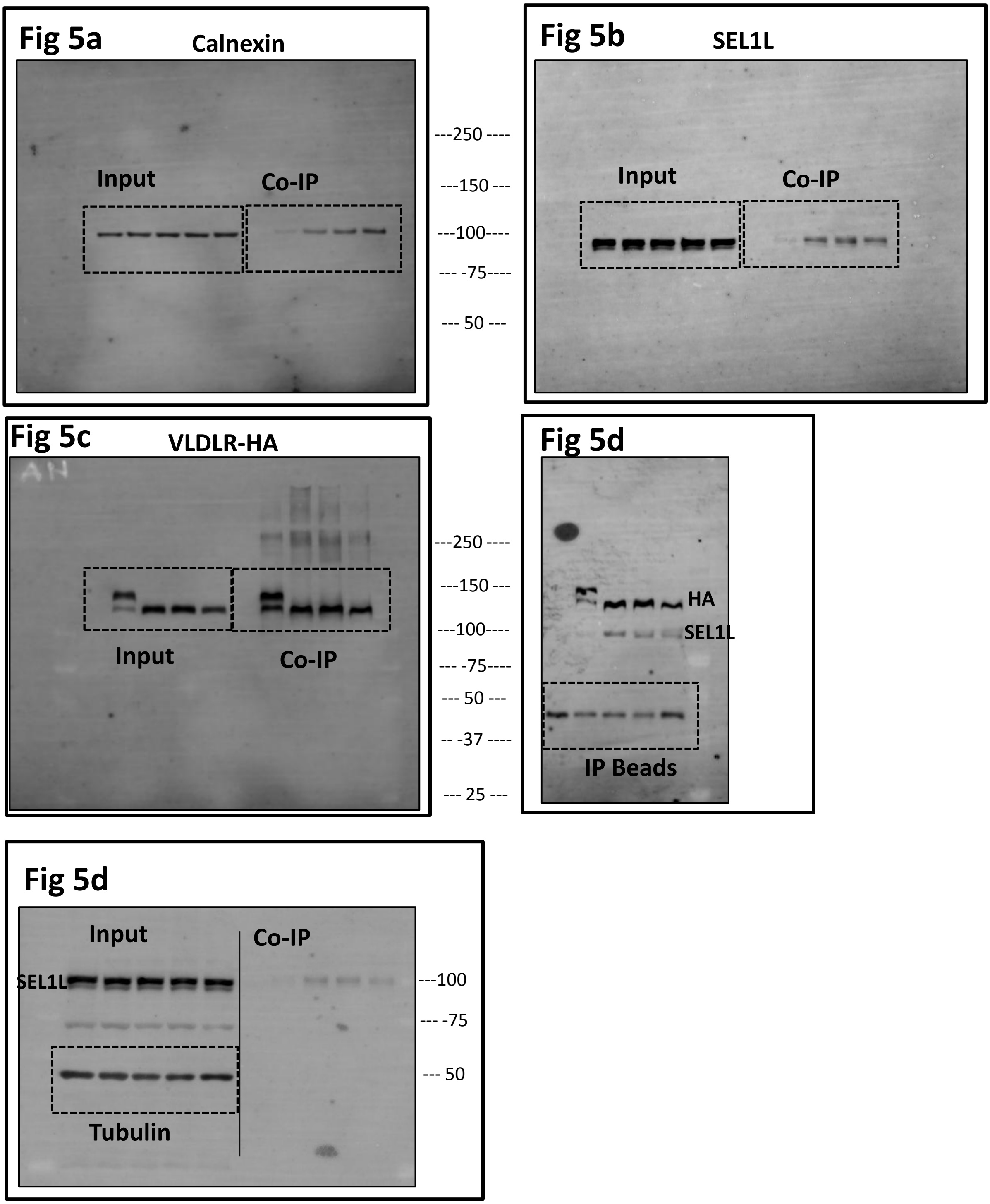

Figure S12: Original scans of western blots displayed on Fig 5a, b, c and d

Figure S13

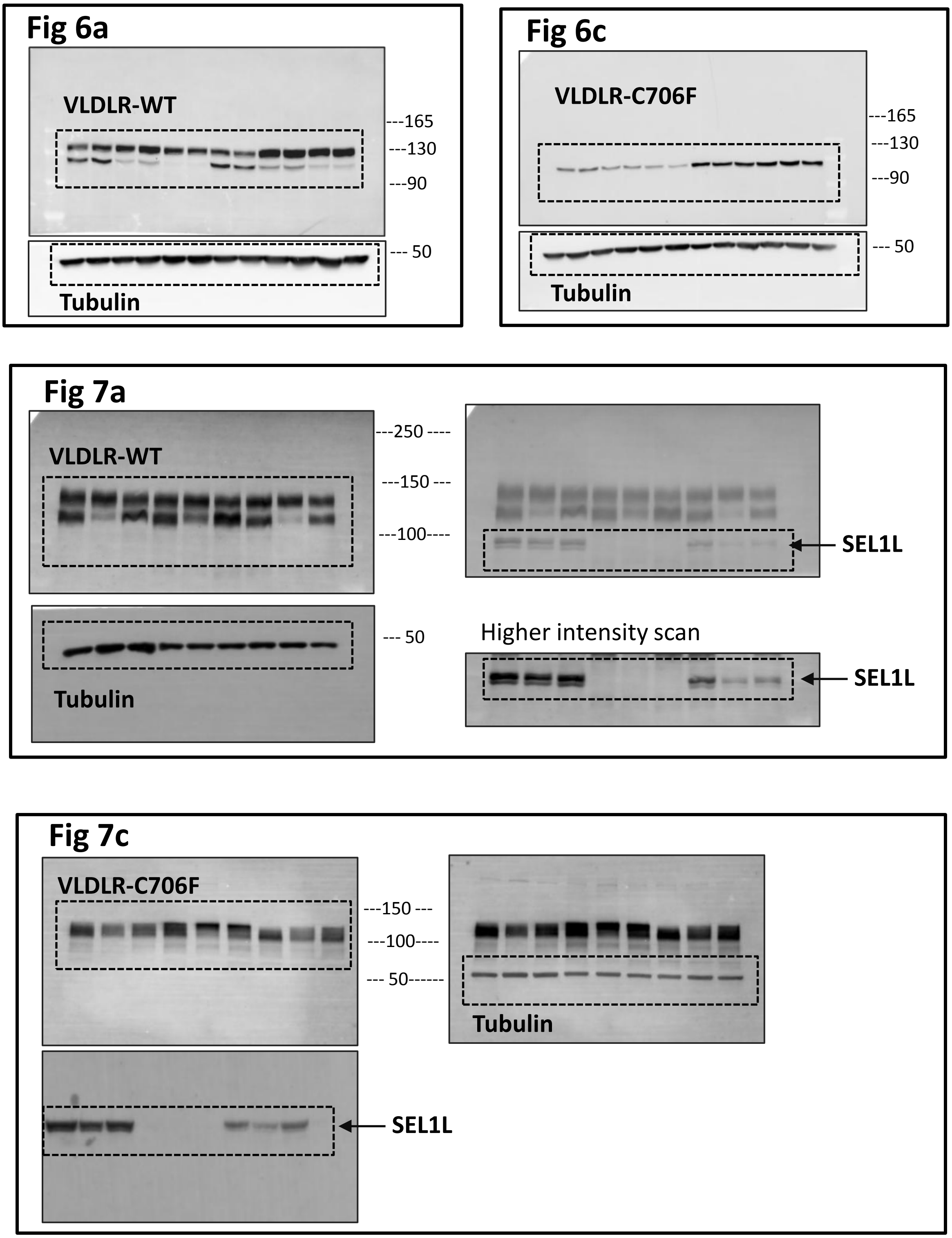

Figure S13: Original scans of western blots displayed on Fig 6a and 6c, Fig 7a and 7c
